# CPVT-associated calmodulin variants N53I and A102V dysregulate Ca^2+^ signalling via different mechanisms

**DOI:** 10.1242/jcs.258796

**Published:** 2022-01-18

**Authors:** Ohm Prakash, Marie Held, Liam F. McCormick, Nitika Gupta, Lu-Yun Lian, Svetlana Antonyuk, Lee P. Haynes, N. Lowri Thomas, Nordine Helassa

**Affiliations:** 1 Liverpool Centre for Cardiovascular Science, Department of Cardiovascular Science and Metabolic Medicine, Institute of Life Course and Medical Sciences, Faculty of Health and Life Sciences, University of Liverpool, Liverpool L69 3BX, UK; 2 Department of Molecular Physiology and Cell Signalling, Institute of Systems, Molecular and Integrative Biology, Faculty of Health and Life Sciences, University of Liverpool, Liverpool L69 3BX, UK; 3 Nuclear Magnetic Resonance Centre for Structural Biology, Institute of Systems, Molecular and Integrative Biology, Faculty of Health and Life Sciences, University of Liverpool, Liverpool L69 7ZB, UK; 4 Molecular Biophysics Group, Institute of Systems, Molecular and Integrative Biology, Faculty of Health and Life Sciences, University of Liverpool, Liverpool L69 7ZB, UK; 5 School of Pharmacy & Pharmaceutical Sciences, Cardiff University, Cardiff, Redwood Building, CF10 3NB, UK

**Keywords:** CPVT, CaMKII, RyR2, Arrhythmia, Calcium signalling, Calmodulin

## Abstract

Catecholaminergic polymorphic ventricular tachycardia (CPVT) is an inherited condition that can cause fatal cardiac arrhythmia. Human mutations in the Ca^2+^ sensor calmodulin (CaM) have been associated with CPVT susceptibility, suggesting that CaM dysfunction is a key driver of the disease. However, the detailed molecular mechanism remains unclear. Focusing on the interaction with the cardiac ryanodine receptor (RyR2), we determined the effect of CPVT-associated variants N53I and A102V on the structural characteristics of CaM and on Ca^2+^ fluxes in live cells. We provide novel data showing that interaction of both Ca^2+^/CaM-N53I and Ca^2+^/CaM-A102V with the RyR2 binding domain is decreased. Ca^2+^/CaM–RyR2_3583-3603_ high-resolution crystal structures highlight subtle conformational changes for the N53I variant, with A102V being similar to wild type (WT). We show that co-expression of CaM-N53I or CaM-A102V with RyR2 in HEK293 cells significantly increased the duration of Ca^2+^ events; CaM-A102V exhibited a lower frequency of Ca^2+^ oscillations. In addition, we show that CaMKIIδ (also known as CAMK2D) phosphorylation activity is increased for A102V, compared to CaM-WT. This paper provides novel insight into the molecular mechanisms of CPVT-associated CaM variants and will facilitate the development of strategies for future therapies.

## Abstract (precis)

**Summary:** CaM mutations associated with human cardiac arrhythmia syndromes result in CaM dysfunction, which appears to be a key driver of the disease, with different mutants acting via different mechanisms.

## INTRODUCTION

Catecholaminergic polymorphic ventricular tachycardia (CPVT) is a genetic channelopathy that usually presents in children and young adults, and can result in sudden death (Behere and Weindling, 2016). CPVT can cause syncope, severe tachyarrhythmia and cardiac arrest under conditions of extreme physical activity or emotional stress in structurally normal hearts (Bardai et al., 2011; Meyer et al., 2012). CPVT has an estimated incidence rate of 1:10,000, with a mortality rate of up to 13% for patients under treatment (Behere and Weindling, 2016). Mutations in the cardiac isoform of the ryanodine receptor (RyR2) are the common genetic basis for CPVT (Lanner et al., 2010; Liu et al., 2017; Priori et al., 2001; Van Petegem, 2012; Zhao et al., 2015); however, recent genetic studies have identified novel calmodulin (CaM) mutations associated with the disease (Chazin and Johnson, 2020; Gomez-Hurtado et al., 2016; Jensen et al., 2018; Makita et al., 2014; Nyegaard et al., 2012; Wang et al., 2020).

In the heart, CaM is a major player in the regulation of various ion channels and pumps (Balshaw et al., 2001; Ben-Johny et al., 2015; Ben-Johny and Yue, 2014; Pitt et al., 2001; Shifman et al., 2006; Van Petegem et al., 2005; Yamaguchi et al., 2003; Zhang et al., 2012). CaM is a highly conserved Ca^2+^-sensing protein encoded by three independent genes *CALM1*, *CALM2* and *CALM3*, and all three encode an identical 16.7 kDa 148 amino-acid-long protein (Fischer et al., 1988; Toutenhoofd et al., 1998). Mutation in any of the six alleles can have critical effects on the excitation-contraction coupling process, and can result in life-threatening arrhythmogenic conditions (Jensen et al., 2018; Nyegaard et al., 2012; Vassilakopoulou et al., 2015). The dumbbell-like structure of CaM comprises of four EF-hand motifs, each able to bind one Ca^2+^ ion. A flexible linker that tethers the N and C globular domains permits high conformational plasticity in binding to targets. Ca^2+^ binding at the two domains is cooperative in nature, with the N-terminal lobe showing faster Ca^2+^ binding compared to the C-terminal lobe (Zhang et al., 2012). The C-terminal lobe has a Ca^2+^ binding affinity that is 6-7 times higher than that of the N-terminal lobe, thus allowing CaM to function either as a rapid or slow Ca^2+^ sensor across a wide range of Ca^2+^ concentrations (Gomez-Hurtado et al., 2016; Hwang et al., 2014; Linse et al., 1991; Sondergaard et al., 2015a,b). Moreover, Ca^2+^ binding induces a large conformational transition in CaM that exposes hydrophobic patches within the helices of the EF-hands, which permit interaction with a wide range of targets.

In cardiomyocytes, during Ca^2+^-induced Ca^2+^ release (CICR), membrane depolarisation activates voltage-gated Ca^2+^ channels (Ca_v_1.2), which causes extracellular Ca^2+^ to enter the cell. The increase in local Ca^2+^ concentrations triggers the release of Ca^2+^ from the sarcoplasmic reticulum (SR) through RyR2 (Dewenter et al., 2017; Endo, 1977). Diffusion of this cytoplasmic Ca^2+^ into the myofibrils promotes the interaction between actin and myosin that results in heart muscle contraction. Tightly controlled cycling of intracellular Ca^2+^ concentration is the basis of normal heart rhythm (Bootman et al., 2002; Landstrom et al., 2017; Luo and Anderson, 2013). The open probability of RyR2 in response to variations in cytoplasmic Ca^2+^ concentration is regulated by both Ca^2+^ free (apo-) and Ca^2+^/CaM (Balshaw et al., 2001, 2002; Brohus et al., 2019; Sigalas et al., 2009; Walweel et al., 2017; Xu and Meissner, 2004; Yamaguchi et al., 2007, 2003; Yang et al., 2014); however, the effect of CPVT-associated CaM mutations on CaM–RyR2 function remains unclear. To determine the molecular mechanism leading to CPVT, a detailed functional, biophysical and structural characterisation of the interaction between CaM and RyR2 is required.

Here, we report the results from a comprehensive analysis of the interaction between CaM CPVT-associated variants – CaM-N53I and CaM-A102V – and the RyR2 CaM-binding domain (CaMBD). The CaM N53I variant was discovered in a large Swedish family with a severe dominantly inherited form of CPVT-like arrhythmias. Using a genome-wide linkage analysis, they demonstrated that the heterozygous missense mutation in the gene encoding calmodulin (*CALM1*) segregated with the disease and showed compromised Ca^2+^ binding (Nyegaard et al., 2012). The CaM A102V variant was identified in *CALM3* in a female who experienced episodes of exertion-induced syncope since 10 years of age, had normal QT interval, and displayed ventricular ectopy during stress testing, consistent with CPVT. CaM-A102V was shown to lower CaM Ca^2+^-binding affinity and promoted spontaneous Ca^2+^ wave and spark activity in permeabilised cardiomyocytes (Gomez-Hurtado et al., 2016).

In this paper, we provide high-resolution three-dimensional structures of CaM variants in complex with RyR2_3583-3603_ and novel data on the binding mechanism of CaM to RyR2. We show for the first time that arrhythmogenic variants N53I and A102V alter intracellular Ca^2+^ oscillation kinetics and can significantly increase CaMKIIδ (also known as CAMK2D) phosphorylation activity. Collectively, these data provide novel insight into the molecular aetiology of CPVT with abnormal Ca^2+^ release from the SR via distinct molecular mechanisms for the N53I and A102V CaM variants.

## RESULTS

### CPVT-associated CaM variants have reduced affinity for RyR2 CaMBD in the presence of Ca^2+^

Both apo- and Ca^2+^/CaM are known to directly bind the RyR2-CaMBD (amino acids 3583-3603) to regulate the gating activity of the channel. To analyse the effect of CPVT-associated mutations on CaM–RyR2_3583-3603_ interaction, we carried out isothermal titration calorimetry (ITC) experiments in the absence (5 mM EGTA) and presence of activating Ca^2+^ concentration (5 mM CaCl_2_). ITC provides value for the dissociation constant (*K*_d_) and stoichiometry of binding (N), and can reveal the nature of the forces that drive the binding reaction (enthalpy change, ΔH and the entropic term ΔS).

**Fig. 1.**
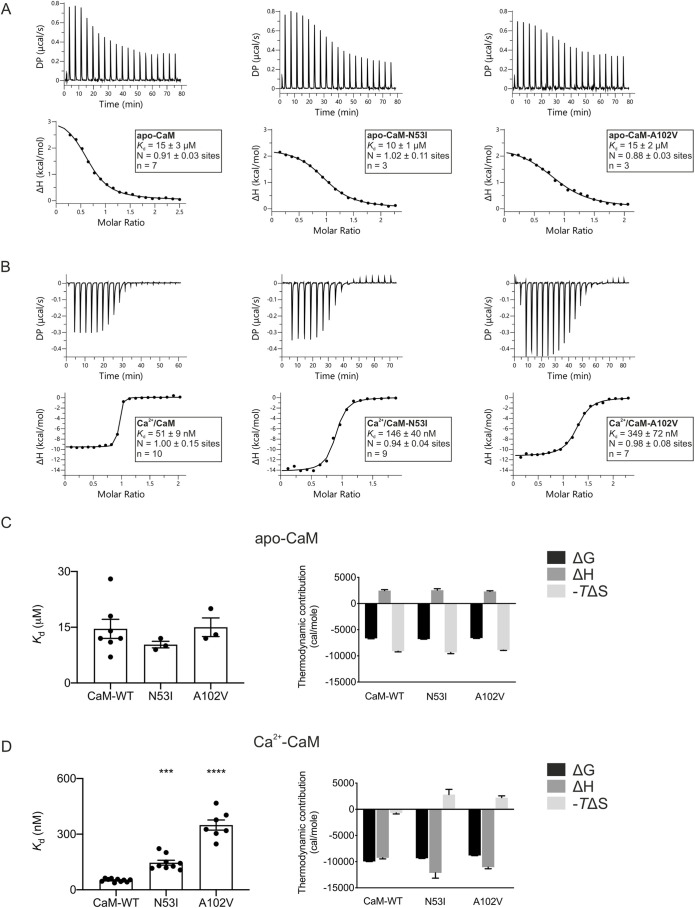
**Ca^2+^/CaM binding to RyR2 is decreased for the CPVT-associated variants N53I and A102V.** (A,B) Representative ITC titration curves (upper panels) and binding isotherms (lower panels) for CaM interaction with RyR2_3583-3603_ in the absence (A) and presence (B) of Ca^2+^. DP, differential power. (C,D) Affinity (left) and thermodynamic profile (right) of the binding of apo-CaM (C) and Ca^2+^/CaM proteins (D) to RyR2_3583-3603_ obtained by fitting to a one-site binding model. Data were processed using the MicroCal PEAQ-ITC software. Data are mean±s.e.m. N, stoichiometry; *n*, number of experimental replicates. The sum of the change in enthalpy (ΔH) and the change in entropy (ΔS) multiplied by the absolute temperature (T) gives the change in free energy (ΔG). Experiments were performed in the presence of 5 mM EGTA or 5 mM CaCl_2_ at 25°C. Number of replicates (*n*) for each condition is shown in the lower panels of A and B. ***P<0.001; ****P<0.0001 (versus CaM-WT; differences between three groups were determined using one-way ANOVA with Dunnett's post-hoc test).

CaM binding to RyR2_3583-3603_ has a stoichiometry of 1:1 in both apo and Ca^2+^ conditions (Fig. 1A,B). In the absence of Ca^2+^, the *K*_d_ of wild-type (WT) CaM for RyR2_3583-3603_ is 15±3 µM (mean±s.e.m.). The CPVT-associated variants (N53I and A102V) did not significantly alter the affinity, with a *K*_d_ of 10±1 µM and 15±2 µM, respectively (Fig. 1C). However, in the presence of Ca^2+^, the affinity for RyR2_3583-3603_ was significantly reduced by up to sevenfold when compared to CaM-WT from 51±9 nM (CaM-WT) to 146±40 nM (CaM-N53I) and 349±72 nM (CaM-A102V) (Fig. 1D).

The Gibbs free energy change (ΔG) for CaM–RyR2_3583-3603_ interaction in both apo and Ca^2+^ conditions was negative which is characteristic of a spontaneous favourable reaction for all CaM variants (Fig. 1C,D). For apo-CaM–RyR2_3583-3603_, the interaction was endothermic and entropy driven (Fig. 1A,C), suggesting an important contribution of hydrophobic interactions. In contrast, the Ca^2+^/CaM–RyR2_3583-3603_ interaction was exothermic and enthalpy driven (Fig. 1B,D). Interestingly, although Ca^2+^/CaM-WT–RyR2_3583-3603_ binding shows favourable entropy, the CPVT variants show unfavourable entropy, indicating a different binding mechanism.

### Secondary structure of CaM is not affected for the CPVT-associated variants

**Fig. 2.**
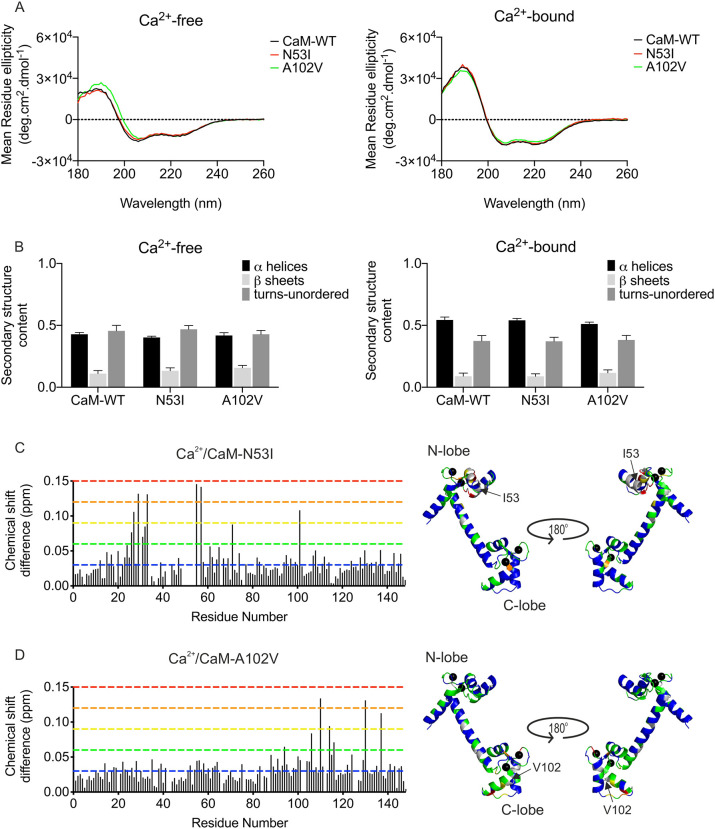
**CPVT-associated CaM variants N53I and A102V do not show altered secondary structure but induce local structural changes in CaM.** Analysis of the secondary structures of CaM-WT and mutants (5 µM) using circular dichroism spectroscopy. (A) Circular dichroism spectra were obtained in the presence of 1 mM EGTA (left panel) or 1 mM CaCl_2_ (right panel). Data are representative traces from experiments performed in triplicate. (B) Protein secondary structure content estimated using the CDSSTR method (Dichroweb, reference set 3). Data are mean±s.e.m. Experiments were performed at 20°C, in triplicate. Differences between the three groups were determined using two-way ANOVA with the Sidak's post-hoc test and CaM-WT as control. No significant differences were found. (C,D) Histogram of chemical shift differences observed between CaM-WT and CaM-N53I (C) or CaM-A102V (D) (left panels). Data shown are from single NMR experiments. Chemical shift data were normalised between minimum and maximum values, and converted into a colour spectrum of five unique colours (key, blue for smallest shifts through to red for largest shifts; shown as dashed lines in histograms), which were then mapped onto the CaM crystal structure (PDB, 2CLL) (right panels). Unassigned peaks are shown in grey, and Ca^2+^ ions as black spheres. Chemical shift differences were expressed in ppm as Δδ=[(ΔH)^2^+(0.15ΔN)^2^]^1/2^. Residues with chemical shifts equal to or less than 0.03 ppm were deemed non-movers (blue); the remaining shifts were categorised into colour by increasing margins of 0.03 ppm (0.03-0.06 ppm, green; 0.06-0.09 ppm, yellow; 0.09-0.12 ppm, orange; and 0.12-0.15 ppm, red). Shift differences were mapped onto the structure of Ca^2+^/CaM using PyMOL to illustrate a surface representation of the chemical shift derivations.

We employed circular dichroism spectroscopy to determine whether CPVT-associated CaM variants induced differences in secondary structure distributions (Fig. 2A,B). In Ca^2+^-free conditions, the α-helical content of CaM-WT was predicted to be 43±1% α-helices and 11±2% β-sheets (mean±s.e.m.). Upon binding to Ca^2+^, the CaM proteins underwent conformational changes with a significant increase in α-helix content (51-54%) measured in all CaM proteins. The CPVT-associated variants did not show significantly altered secondary structure content of CaM in apo- or Ca^2+^-saturating conditions compared to the WT protein.

### N53I and A102V CaM variants show altered protein stability

To determine whether activating levels of Ca^2+^ and/or RyR2 binding induced significant conformal changes that would affect susceptibility to proteases, we employed a biochemical assay using the endoproteinase AspN (Fig. S1A,B). In apo conditions, we observed that over 97% of CaM proteins were degraded when incubated with 0.6 μg ml^−1^ AspN (Fig. S1A). Complex formation between apo-CaM and RyR2_3583-3603_ induced a significant resistance to protease cleavage, with 5.0 μg ml^−1^ required to degrade over 90% of WT and A102V CaM proteins. The apo-CaM-N53I–RyR2_3583-3603_ variant exhibited a substantial increase in stability, requiring 10.0 μg ml^−1^ of AspN to achieve similar levels of degradation as the apo-CaM-WT–RyR2_3583-3603_ and apo-CaM-A102V–RyR2_3583-3603_ complexes.

Ca^2+^/CaM proteins showed a significant reduction in susceptibility to AspN cleavage with 500× more AspN protease (300 μg ml^−1^) required to achieve full degradation compared to apo-CaM. Ca^2+^/CaM-N53I showed an increased susceptibility to AspN degradation compared to the A102V and WT forms (Fig. S1B). Complex formation of Ca^2+^/CaM with RyR2_3583-3603_ again increased the stability of CaM, with only 19±2% (mean±s.e.m.) of the WT degraded at high AspN concentrations (300 μg ml^−1^), and the CPVT-associated variants did not induce any significant differences.

Interestingly, despite the susceptibility to protease degradation remaining unchanged across the apo-CaM species, we observed that the CPVT-associated variants have significantly reduced temperature stability. The melting temperature (T_m_) assessed by circular dichroism decreased from 41.6±0.3°C (mean±s.e.m.) to 37.0±0.7°C (N53I) and 39.3±0.4°C (A102V) (Fig. S1C).

### CPVT-associated mutations N53I and A102V induce subtle local structure perturbations

Two-dimensional ^1^H-^15^N heteronuclear single quantum coherence nuclear magnetic resonance (HSQC NMR) was used to investigate the effect of the mutations on the three-dimensional structure of Ca^2+^/CaM. We were able to unambiguously assign 144 of 148 (minus two proline residues, Asn42 and Met76) backbone resonances for Ca^2+^/CaM-WT. Over 92% of WT resonance assignments were transferred to both CaM-N53I and CaM-A102V HSQC spectra.

Comparison of chemical shifts between CaM-WT and CaM-N53I spectra revealed, as expected, significant perturbations of chemical shifts around the site of amino acid substitution, with the largest shift difference occurring at Val55 (0.146 ppm; Fig. 2C). The residues Thr26-Gly33 and Val55-Ala57 showed noticeable chemical shifts, with additional shift changes detected for more distal residues Met71 and Ser101.

On the other hand, the shift differences of CaM-A102V revealed a more localised effect when the shift perturbations (A102V versus WT) were mapped onto the protein structure (Fig. 2D). The residues with large chemical shifts were confined to the C-terminal region and were as follows: Lys94, Arg106, Thr110, Glu114, Leu116, Ile130 and Asn137. Most differences were observed around the site of mutation located at the third EF-hand motif (largest shift changes in Thr110, 0.135 ppm), with fewer residues substantially perturbed at the neighbouring Ca^2+^-binding motif. Despite variations in the chemical shift perturbations in the two variants, larger chemical shift changes appeared to localise within the respective lobe at which they occurred. This indicates that the CPVT-associated mutations do not globally remodel protein structure but induce localised changes to protein architecture, particularly at the sites responsible for Ca^2+^ co-ordination.

### The C-lobe of Ca^2+^/CaM forms the initial site of interaction with RyR2

Using two-dimensional ^1^H-^15^N HSQC NMR and stepwise titration of the RyR2_3583-3603_ peptide to Ca^2+^/CaM, we were able track the changes in chemical shifts and deduce structural information about how the complexes between CaM and RyR2 are formed. At 1 M equivalent peptide, 46% of the backbone resonances were assigned; the same percentage of residues were also assignable for the CaM-N53I and CaM-A102V variants.

Upon the addition of 1 M equivalent RyR2_3583-3603_ peptide to CaM-WT, residues from the C-lobe were twice as likely to be affected by the presence of the peptide than the N-lobe residues (Table S1). Only 29% of the resonances from the C-lobe compared with 57% from the N-lobe, were not affected by the presence of the peptide. Interestingly, when the RyR2_3583-3603_ peptide was added to 2 M equivalents, we observed the opposite effect: 58% and 92% of the resonances in the N- and C-lobes, respectively, were not significantly perturbed. Therefore, the C-lobe residues appear to be more affected at lower concentrations of the peptide, whereas N-lobe residues respond when the peptide is at a higher concentration. The same chemical shift perturbation phenomenon is also observed for CaM-N53I and CaM-A102V. These data suggest that in all three Ca^2+^/CaM forms, the C-lobe forms the initial site of interaction.

From the titration experiments, CaM-N53I and CaM-A102V did not result in altered complex formation with the RyR2_3583-3603_ peptide. Binding of the peptide occurs via the C-lobe initially, followed by N-lobe conformational changes as evidenced by the chemical shifts analysis.

### Crystal structure of Ca^2+^/CaM-N53I–RyR2_3583-3603_ shows subtle conformational changes when compared to that of WT

**Fig. 3.**
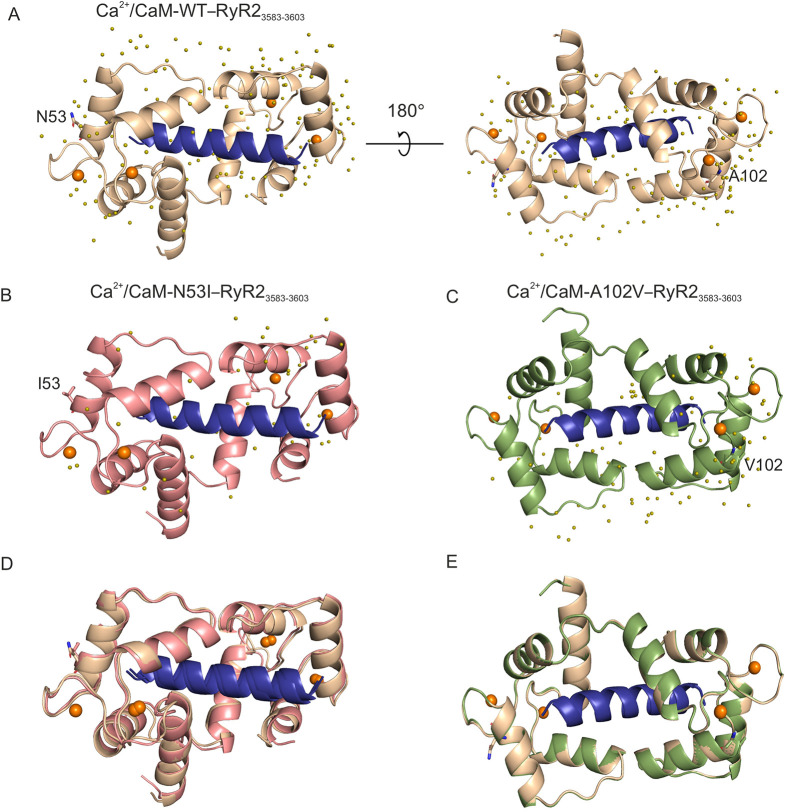
**The arrhythmogenic N53I mutant causes subtle changes in the three-dimensional structure of Ca^2+^/CaM–RyR2_3583-3603_.** (A-C) Cartoon representation of the crystal structures of Ca^2+^/CaM proteins in complex with RyR2 peptide. (A) Ca^2+^/CaM-WT–RyR2_3583-3603_ (PDB 6XXF). (B) Ca^2+^/CaM-N53I–RyR2_3583-3603_ (PDB 6XY3). (C) Ca^2+^/CaM-A102V–RyR2_3583-3603_ (PDB 6XXX). (D,E) Alignments of Ca^2+^/CaM-WT–RyR2_3583-3603_ with Ca^2+^/CaM-N53I–RyR2_3583-3603_ (D) or Ca^2+^/CaM-A102V–RyR2_3583-3603_ (E) complex structures. Ca^2+^ ions are shown as orange spheres and water molecules as olive spheres. The WT and mutant residues are shown in stick representation. CaM-WT is displayed in beige, CaM-N53I in salmon, CaM-A102V in green and RyR2_3583-3603_ peptide in blue. Images were generated using PyMOL software.

We co-crystallised Ca^2+^/CaM variants with the RyR2_3583-3603_ peptide to gain an atomistic understanding of the interaction. Ca^2+^/CaM-WT–RyR2_3583-3603_ [Protein Data Bank (PDB), 6XXF], Ca^2+^/CaM-N53I–RyR2_3583-3603_ (PDB, 6XY3) and Ca^2+^/CaM-A102V–RyR2_3583-3603_ (PDB, 6XXX) crystal structures were solved to a high resolution of 1.70Å, 2.00Å and 1.25Å, respectively (Fig. 3A-C). Crystallographic data and refinement statistics are shown in Table S2. All the complexes gave clear electron density for four Ca^2+^ ions at the N- and C-terminal Ca^2+^ binding grooves. To determine the effect of CPVT-associated mutations on the structure of Ca^2+^/CaM–RyR2_3583-3603_ complexes, we compared Ca^2+^/CaM-WT–RyR2_3583-3603_ with Ca^2+^/CaM-N53I–RyR2_3583-3603_ and Ca^2+^/CaM-A102V–RyR2_3583-3603_ peptide complex crystal structures obtained in this study (Fig. 3D,E). The interactions between CaM residues and Ca^2+^ ions were conserved and identical in all the structures. In addition to the overall structural superimposition, we superimposed individual regions of the Ca^2+^/CaM–RyR2_3583-3603_ peptide complex onto specific domains to identify the region with considerable structural root mean square deviation (RMSD) (Fig. S2A,B, Table S3). The overall structure of all the compared complexes showed similar conformation to the Ca^2+^/CaM-WT–RyR2_3583-3603_ peptide complex. Based on the RMSD values, the flexible helix region seems to contribute the most towards the observed small structural discrepancy.

### CPVT mutations induce subtle changes in hydrophobic, hydrogen and salt-bridge interactions in Ca^2+^/CaM–RyR2 structures

**Fig. 4.**
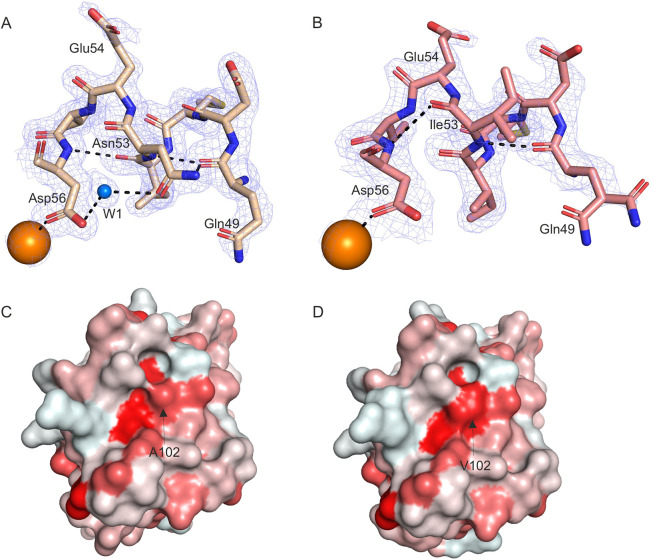
**Ca^2+^/CaM–RyR2_3583-3603_ peptide complexes show differences in the H-bond network at the site of mutation, and increased hydrophobicity.** (A,B) Ca^2+^/CaM-WT–RyR2_3583-3603_ with Ca^2+^/CaM-WT in beige (A) and Ca^2+^/CaM-N53I–RyR2_3583-3603_ with Ca^2+^/CaM-N53I in salmon (B). Ca^2+^ ions are shown as orange spheres and water molecule as a blue sphere. Interactions are represented by black dashed lines, with residues represented as sticks and electron density as mesh. (C,D) Comparison of the hydrophobicity of the surface areas of Ca^2+^/CaM-WT–RyR2_3583-3603_ (C) and Ca^2+^/CaM-A102V–RyR2_3583-3603_ (D). CaM is shown in surface representation, with surface coloured from white to red based on the Eisenberg hydrophobicity scale (Eisenberg, 1984). White is less hydrophobic than red. Arrows indicate the areas of the Ala102 and Val102 residues. Images were generated using PyMOL software.

The Ca^2+^/CaM-WT–RyR2_3583-3603_ and Ca^2+^/CaM-N53I–RyR2_3583-3603_ peptide complex structure show subtle differences in the mutated region (Fig. 4A,B). However, these differences do not result in major structural differences in RyR2_3583-3603_ peptide binding (Table S3, RMSD 1.31Å), with the most prominent difference being the side chain orientation of amino acid Glu54 and the disruption of the hydrogen-bonding (H-bond) network near Ile53 (Fig. 4A,B). In the Ca^2+^/CaM-WT–RyR2_3583-3603_ structure, atoms N^δ2^ Asn53 and N Asn53 form a direct hydrogen bond with O of another CaM residue, Gln49. O^δ1^ Asn53 also forms a hydrogen bond with O^δ2^ Asp56 through the water molecule W1. Asp56 in turn is involved in Ca^2+^ binding in the EF-hand (Fig. 4A). In Ca^2+^/CaM-N53I–RyR2_3583-3603_, the interactions of the main chain atoms N Ile53 and O Gln49 are retained. However, as isoleucine (Ile/I) is a hydrophobic amino acid in contrast to asparagine (Asn/N), which is polar hydrophilic, the water molecule W1, which forms part of the H-bond network, is lost in the N53I mutant CaM. Interestingly, O Ile53 then forms a direct hydrogen bond with N Asp56 (Fig. 4B). We observed that the Ca^2+^/CaM-A102V–RyR2_3583-3603_ complex is structurally very similar to Ca^2+^/CaM-WT–RyR2_3583-3603_ (Fig. 3E; Fig. S2B, Table S3, RMSD 0.63Å). Even though no major structural differences could be discerned at the vicinity of the mutation, the A102 V substitution increases interaction surface hydrophobicity compared with Ca^2+^/CaM-WT–RyR2_3583-3603_ structure (Fig. 4C,D).

The H-bond and salt-bridge interactions between the Ca^2+^/CaM and the corresponding peptides were predicted by the PDBePISA server (Krissinel and Henrick, 2007). Although most of the interactions are conserved in all the complexes, subtle differences in the side chain orientations change the individual atoms involved in the interactions (Table S4). Residues from Ca^2+^/CaM and corresponding peptides involved in H-bonds and salt bridges unique to each are represented in Fig. S3.

### CPVT-associated CaM variants N53I and A102V differentially modulate Ca^2+^/CaM kinase II phosphorylation activity

**Fig. 5.**
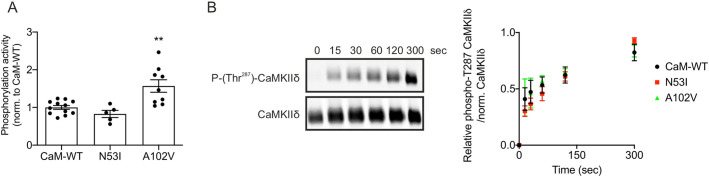
**Arrhythmogenic variant A102V increases CaMKIIδ substrate phosphorylation activity, whereas T287 CaMKIIδ autophosphorylation levels remain unchanged.** (A) Quantification of phosphorylation activity of CaMKIIδ using radiolabelled ATP. CaMKIIδ activity measurements were performed using the SignaTECT Calcium/Calmodulin-Dependent Protein Kinase Assay System (Promega) on freshly isolated mice hearts. CaM-WT, CaM-N53I or CaM-A102V recombinant proteins (1 µM) were used as CaMKIIδ activators. [γ-^32^P]-syntide-2 was used as an indicator of CaMKIIδ phosphorylation activity and quantified using a scintillation counter. The numbers of experimental replicates were as follows: *n*=12 for CaM-WT, *n*=5 for CaM-N53I and *n*=9 for CaM-A102V, from three independent mice heart lysate preparations. Data are mean±s.e.m. **P<0.01 (versus CaM-WT; differences between the three groups were determined using one-way ANOVA with Dunnett's post-hoc test). (B) Measurement of the relative levels of CaMKIIδ Thr^287^ autophosphorylation. GST-CaMKIIδ was incubated with calmodulin variants and ATP for 0 s, 15 s, 30 s, 60 s, 120 s and 300 s at room temperature. CaM-WT, CaM-N53I or CaM-A102V recombinant proteins were used as CaMKIIδ activators. The reaction was terminated using SDS-containing Laemmli buffer, and samples were analysed by western blotting and densitometry analysis. Representative blots show CaM-N53I samples. Phosphorylated proteins (phospho-Thr^287^ antibody) were normalized to total CaMKIIδ protein (GST antibody). Data are mean±s.e.m. The numbers of experimental replicates were as follows: *n*=4 for CaM-WT, *n*=3 for CaM-N53I and *n*=3 for CaM-A102V.

Using radiolabelled ATP and mice heart lysate as a source of Ca^2+^/CaM kinase II (CaMKIIδ), phosphorylation levels of syntide-2 (CaMKIIδ substrate) were measured using WT and CPVT-associated CaM variants (SignaTECT, Promega). We showed that, when using CaM-N53I as a CaMKIIδ activator, phosphorylation of syntide-2 remained unaffected, whereas CaM-A102V significantly increased phosphorylation levels by 57±17% (mean±s.e.m.), compared to CaM-WT (Fig. 5A).

Using commercial human CaMKIIδ recombinant protein (Abcam, ab84552), the rate of Thr^287^ autophosphorylation for Ca^2+^/CaM–CaMKIIδ was estimated by western blotting and densitometry analysis (Fig. 5B). We did not observe a significant difference between CaM-WT and disease-associated mutants, demonstrating that the increase in phosphorylation activity observed for CaM-A102V cannot be attributed to enhanced autophosphorylation.

### CaM-N53I and A102V alter spontaneous Ca^2+^ signalling events in cells

**Fig. 6.**
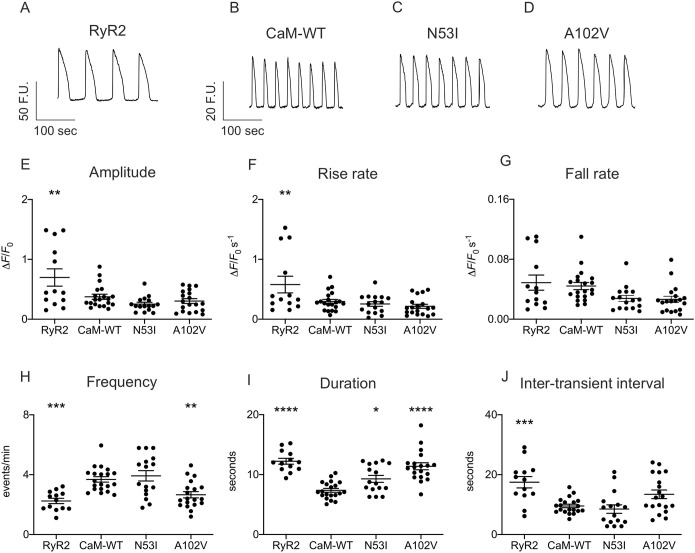
**CPVT-associated mutations N53I and A102V affect spontaneous Ca^2+^ transients in cells.** HEK293T cells transfected with RyR2 and CaM variants were loaded with Calbryte 520 to monitor intracellular Ca^2+^ concentration changes. Live cells were imaged on a 3i spinning-disk confocal microscope. (A-D) Representative fluorescence signals in HEK293T cells expressing RyR2 (A) with CaM-WT (B), CaM-N53I (C) and CaM-A102V (D). (E-J) Analysis of the Ca^2+^ transients kinetic parameters using Analyse Spikes applet for Matlab and Fiji (Schindelin et al., 2012). Data are mean±s.e.m. The numbers of experimental replicates for the oscillations experiments (*N*=dishes, *n*=fields of view) were as follows: *N*=6, *n*=20 for CaM-WT; *N*=6, *n*=16 for CaM-N53I; and *N*=6, *n*=19 for CaM-A102V. *P<0.05; **P<0.01; ***P<0.001; ****P<0.0001 (versus CaM-WT; differences between the groups were determined using one-way ANOVA with Dunnett's post-hoc test). F.U., fluorescence unit; Δ*F*, difference between the maximum fluorescence and the initial fluorescence; *F*_0_, initial fluorescence.

To assess the effect of CPVT-associated CaM mutations on Ca^2+^ release from the endoplasmic reticulum (ER), we transiently overexpressed human RyR2 and CaM variants in HEK293T cells. Using Calbryte 520 as a Ca^2+^ indicator, we measured spontaneous Ca^2+^ oscillations using single-cell fluorescence confocal microscopy. Cells that exhibited regular spontaneous Ca^2+^ release events, indicative of functional RyR2 expression (Jiang et al., 2004), were included in the analysis of kinetic parameters (Fig. 6). Co-expression of CaM-WT with RyR2 decreased the amplitude of Ca^2+^ transients and affected their duration, making them significantly shorter, to the extent that the frequency of events was increased, with the interval between release events (inter-transient interval) shorter than that in cells expressing RyR2 alone.

CPVT-associated CaM mutations did not affect amplitude, inter-transient interval, ER load, rise and fall rate of RyR2-mediated Ca^2+^ release events compared to CaM-WT. However, CaM-N53I and A102V resulted in a significant increase in the duration of the events, which significantly reduced the frequency for CaM-A102V (Fig. 6; Fig. S4A).

We did not observe any significant effect of the CaM mutations on intracellular Ca^2+^ concentrations and non-RyR-associated Ca^2+^ responses when stimulated with the muscarinic receptor agonist carbachol or the SERCA inhibitor thapsigargin (Fig. S4B-D).

## DISCUSSION

CaM is an ubiquitously expressed Ca^2+^ sensor that regulates several target proteins involved in excitation-contraction coupling in the heart, including RyR2 and Ca_v_1.2 (Balshaw et al., 2001; Hoeflich and Ikura, 2002; Kim et al., 2004; Lanner et al., 2010; Pitt et al., 2001; Yamaguchi et al., 2003; Yang et al., 2003). CaM modulates RyR2-mediated Ca^2+^ release from the SR either via direct inhibition of RyR2 or indirectly via phosphorylation of RyR2 by CaMKIIδ (Ai et al., 2005; Walweel et al., 2019, 2017). Finely tuned CaM-RyR2 interaction is essential for normal SR Ca^2+^ release and heart function. Abnormal interaction between CaM and RyR2 due to mutations in the CaMBD of RyR2 or in CaM itself can cause severe cardiac complications, including cardiac hypertrophy, heart failure and arrhythmias, such as CPVT (Balshaw et al., 2001; Yamaguchi et al., 2013, 2007). In this paper, we used a multidisciplinary approach combining protein biophysics, structural biology and Ca^2+^ imaging to determine the effect of the CPVT-associated CaM variants N53I and A102V on the CaM–RyR2 structure-function relationship. The major biophysical properties of CPVT-associated CaM variants in comparison to CaM-WT, determined in this study, are summarized in Table 1.

**Table 1.**
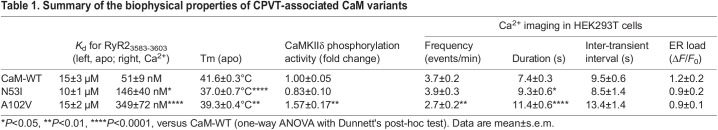
Summary of the biophysical properties of CPVT-associated CaM variants

Disease-associated mutations can affect protein folding and/or reduce protein stability, leading to protein dysfunction. In accordance with previous studies investigating CaM-N53I, we showed that the secondary structure of the CaM-N53I variant was unchanged (Sondergaard et al., 2015a; Vassilakopoulou et al., 2015), and observed a reduced thermal stability (Sondergaard et al., 2015a) compared to CaM-WT. We demonstrated that conformational change adopted by CaM in the presence of Ca^2+^ and/or RyR2 protected the protein against protease degradation (AspN), suggesting a significant change in the local intramolecular dynamics of the protein. In all conditions tested, A102V susceptibility to proteases was similar to CaM-WT, indicating that only the N53I variant induced subtle but notable structural changes.

Using ITC, we determined, for the first time, the binding and thermodynamic properties of CaM A102V–RyR2_3583-3603_. We showed that the interaction of apo-CaM-WT with RyR2_3583-3603_ is entropy driven (hydrophobic interaction), with a *K*_d_ of 15±3 µM (mean±s.e.m.). In the absence of Ca^2+^, the CPVT-associated variants N53I (Holt et al., 2020) and A102V did not significantly affect the affinity. For Ca^2+^/CaM, interaction with RyR2_3583-3603_ is enthalpy driven and *K*_d_ is significantly decreased from 15±3 µM to 52±4 nM, as previously shown (Holt et al., 2020; Lau et al., 2014). In contrast to other studies, which did not show any difference in the binding affinity of Ca^2+^/CaM-N53I and Ca^2+^/CaM-A102V for RyR2 (Gomez-Hurtado et al., 2016; Holt et al., 2020), we observed a significant threefold and sevenfold decrease in affinity for N53I and A102V, respectively. Using similar experimental conditions to Holt et al. (2020) (longer RyR2 peptide RyR2_3581-3608_ and absence of magnesium), we still observed significant reduced affinities for the disease-associated CaM mutants (Fig. S5).

Ca^2+^/CaM-WT–RyR2_3583-3603_ binding showed favourable entropy, suggesting an important contribution of hydrophobic interactions, whereas the CPVT variants have unfavourable entropy, indicating a different binding mechanism comprising hydrogen and Van der Waals bonds, which can account for the measured reduction in affinity. Upon Ca^2+^ binding, CaM undergoes a conformational switching that drives target binding. Interestingly, it has been shown that the N53I mutation does not significantly alter Ca^2+^ affinity (Hwang et al., 2014; Vassilakopoulou et al., 2015), whereas CaM-A102V has a threefold larger *K*_d_ for Ca^2+^ (Gomez-Hurtado et al., 2016). The impaired Ca^2+^ binding can affect CaM structural transitions and thus subsequent interactions with its target RyR2. Altogether, our data indicate that in regard to the physiology of CPVT, pre-association of CaM to RyR2 is not altered; however, when intracellular Ca^2+^ increases during CICR, the decreased affinity for RyR2 reduces the inhibition of the channel and can promote Ca^2+^ release from the SR.

In CaM-associated CPVT syndromes, only one out of six CaM alleles are mutated. However, it has been shown that in the presence of a threefold excess of CaM-WT, the variants CaM N53I and CaM-A102V can promote significantly higher Ca^2+^ wave frequencies in permeabilized cardiomyocytes compared to CaM-WT (Gomez-Hurtado et al., 2016; Hwang et al., 2014). These data demonstrate that CPVT-associated CaM mutations can lead to a dominant pathogenic effect, which is consistent with an autosomal dominant inheritance pattern in humans. The functional dominance can be explained by the fact that the tetrameric RyR2 Ca^2+^ channel has four CaM-binding sites, and binding of only one single mutant CaM may be sufficient to disrupt the CaM-dependent regulation of Ca^2+^ release from the SR.

We investigated the dynamics and atomistic details of Ca^2+^/CaM–RyR2_3583-3603_ interaction by NMR spectroscopy and x-ray crystallography. This revealed for the first time the stages of Ca^2+^/CaM–RyR2_3583-3603_ complex formation through two-dimensional ^1^H-^15^N HSQC NMR. The binding event is initiated by the C-terminal region of CaM and further completed by the N-terminal region. This dynamic characteristic of Ca^2+^/CaM–RyR2_3583-3603_ peptide complex formation is conserved across all the CaM variants (WT, N53I and A102V).

The NMR chemical shift perturbations of the variants show that in addition to the residues that are sequentially and spatially close to the mutated residues being affected, more spatially distant residues also undergo shift perturbations. This indicates some intramolecular structural rearrangement of the tertiary structures in the variants.

We deposited high-resolution structures for Ca^2+^/CaM-WT bound to RyR2_3583-3603_ (PDB 6XXF), Ca^2+^/CaM-N53I–RyR2_3583-3603_ (PDB 6XY3) and Ca^2+^/CaM-A102V–RyR2_3583-3603_ (PDB 6XXX). Superimposition of Ca^2+^/CaM-WT–RyR2_3583-3603_ onto Ca^2+^/CaM-N53I–RyR2_3583-3603_ showed subtle structural variations, whereas Ca^2+^/CaM-A102V–RyR2_3583-3603_ is more similar to the WT structure (lower structural RMSD between individual domains). The largest structural RMSD was observed at the linker helix domain of CaM, with some of the residue side chains not fully resolved in the x-ray structures due to their enhanced flexibility. This region is known to be highly flexible and plays a role in the structural transition of CaM upon Ca^2+^ and target binding. The hydrophobic nature of the mutant residues causes changes with respect to the H-bond network and side-chain orientations at the site of mutation. As Ile has a larger hydrophobicity index value than Val residue (1.38>1.08) (Eisenberg, 1984), these differences are more prominent in CaM-N53I than in CaM-A102V. These localised changes did not affect the peptide binding or the overall architecture of the Ca^2+^/CaM–RyR2_3583-3603_ complex, which is in agreement with the recent structures deposited by Holt et al. (2020). However, intramolecular dynamic changes within the local environment at the site of mutation could affect the functional outcome of the interaction. The H-bond and salt-bridge interactions between the CaM variants and RyR2_3583-3603_ peptide showed subtle differences. We observed unique H-bond interactions in the Ca^2+^/CaM-N53I–RyR2_3583-3603_ complex involving residues Glu84, Leu39 from CaM with Arg3597, and Arg3595 from RyR2. Since electron density for the side chain of Glu123 and Lys75 was not present in the x-ray structure of Ca^2+^/CaM-N53I–RyR2_3583-3603_, the interaction of these residues could not be deciphered. The salt-bridge interactions in Ca^2+^/CaM-N53I–RyR2_3583-3603_ showed no differences compared to the Ca^2+^/CaM-WT–RyR2_3583-3603_ complex. For the Ca^2+^/CaM-A102V–RyR2_3583-3603_ complex, the H-bond network is more similar to Ca^2+^/CaM-WT–RyR2_3583-3603_, with just a single extra interaction (Met144-His3588) and a single lost interaction (Asn111-Arg3597). In the Ca^2+^/CaM-A102V–RyR2_3583-3603_ complex, Lys148 forms an extra salt-bridge interaction with His3588 in RyR2, and the interaction of residue Glu11 with Arg3595 in RyR2 is absent.

Phosphorylation of the RyR2 channel is an important prerequisite for the inhibitory CaM regulation in healthy human hearts (Walweel et al., 2019). One of the major kinases involved in RyR2 channel phosphorylation is CaMKIIδ (Edman and Schulman, 1994; Hoch et al., 1999; Hund and Mohler, 2015). Phosphorylation of RyR2 channel by CaMKIIδ subsequently escalates the RyR2-mediated SR Ca^2+^ leak and delayed afterdepolarizations (Liu et al., 2011; Wehrens et al., 2004). CaMKIIδ is a multifunctional Ser/Thr protein kinase that modulates RyR2 and Ca_v_1.2 activity. Upon Ca^2+^-CaM activation, this multimeric holoenzyme can first autophosphorylate and then phosphorylate target channels to regulate their function (Hudmon et al., 2005; Lanner et al., 2010; Lucic et al., 2008; Rodriguez et al., 2003; Wehrens et al., 2005, 2004; Witcher et al., 1991, 1992). It has been shown using the genetically encoded sensor ‘Camui’ that CaMKII activity was decreased in idiopathic ventricular fibrillation and long QT syndrome (Berchtold et al., 2016; Hwang et al., 2014). However, very limited information exists on the effect of CaM mutations on CaMKIIδ activity in CPVT. Using radiolabelled [γ^32^P] ATP and syntide-2 as a model CaMKIIδ substrate, we showed that Ca^2+^/CaM-N53I did not affect the kinase action of CaMKIIδ, as previously shown (Berchtold et al., 2016). However, we showed for the first time that Ca^2+^/CaM-A102V significantly increased the substrate phosphorylation levels by ∼60%. These novel data suggest a distinct mechanism of RyR2 channel inhibitory action by CaM-N53I and CaM-A102V in CPVT, potentially via differential CaMKIIδ activation.

CPVT-associated CaM mutations have been shown to evoke arrhythmogenic Ca^2+^ waves in cardiomyocytes (Gomez-Hurtado et al., 2016; Hwang et al., 2014). In HEK293 cells expressing murine RyR2, Sondergaard et al. measured the effect of various arrhythmogenic CaM variants on ER Ca^2+^ concentration using the fluorescence resonance energy transfer probe D1ER. They showed that CaM variants, such as A102V, can decrease RyR2 activation/termination thresholds during store-overload-induced Ca^2+^ release (Sondergaard et al., 2019). In addition, it has been shown that N53I and A102V variants increased RyR2 open probability (P*o*) (Sondergaard et al., 2019, 2016). However, the effect of the CaM variants on the Ca^2+^ oscillation kinetic parameters, as we demonstrate in this study, was not investigated. To determine the effect of the N53I and A102V CaM mutations on RyR2 function and Ca^2+^ fluxes, we performed a detailed analysis of spontaneous Ca^2+^ release events in HEK293T cells co-expressing human RyR2 and the CaM variants. Co-expression of CaM-WT with RyR2 decreased the amplitude of spontaneous Ca^2+^ signalling events and reduced their duration and the inter-transient interval compared to cells expressing RyR2 alone. This is consistent with an inhibitory effect of CaM on the channel, decreasing the channel open time (Xu and Meissner, 2004). As this represents a partial reversion of the Ca^2+^ release phenotype to that in the absence of CaM co-expression (i.e. RyR2 alone), this suggests that CPVT-associated mutations induce a loss of inhibitory action of the channel. In this study, we provide novel data showing a significant increase in the duration of the Ca^2+^ events for both CaM-N53I and CaM-A102V, resulting in a reduced frequency for A102V. Therefore, the CPVT-associated CaM variants N53I and A102V not only increase the RyR2 P*o* but also cause changes in Ca^2+^ release dynamics at a global cellular level, which will contribute to the arrhythmia phenotype.

We did not observe any significant effect of the CaM mutations on intracellular Ca^2+^ concentrations and non-RyR-associated Ca^2+^ response (carbachol or thapsigargin), suggesting that CaM effects on Ca^2+^ signalling are essentially RyR2 mediated.

The CaM N53I variant has been identified in a large Swedish family in which the mutation has been carried over for generations (Nyegaard et al., 2012). It represents the largest cohort of patients living with an arrhythmia-associated CaM mutation, which suggests that CaM N53I has a less severe clinical phenotype than other *de novo* CaM variants, such as CaM A102V. This observation correlates with our data that showed a more pronounced dysregulation of Ca^2+^ signalling pathways for CaM A102V.

**Fig. 7.**
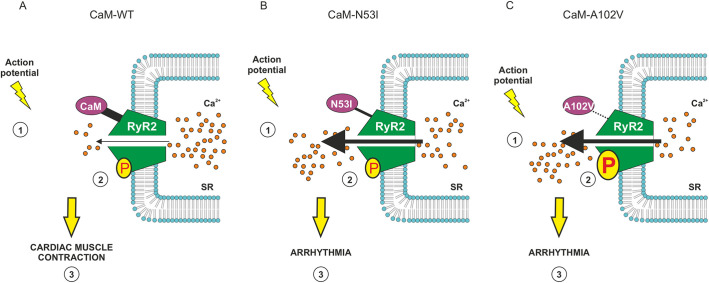
**Proposed regulatory mechanisms for CPVT-associated CaM variants.** (1) Upon stimulation (action potential), Ca^2+^ enters the cell through L-type Ca^2+^ channels (Ca_v_1.2) and (2) triggers release from the RyR2 by a process known as CICR. (3) Cytosolic Ca^2+^ diffuses to myofibrils and promotes interaction between actin and myosin that leads to cardiac muscle contraction. (A) In normal conditions, Ca^2+^/CaM modulates RyR2 activity through direct binding (inhibition) and phosphorylation (P) via CaMKII (activation). A fine-tuned balance between these two processes is required to control cardiac muscle contraction. (B) CaM-N53I: a moderate reduction in affinity for RyR2 and altered CaM conformation reduce the inhibition of RyR2 by Ca^2+^/CaM. This leads to an increase of Ca^2+^ release from the SR, promoting arrhythmia. (C) CaM-A102V: a large reduction in affinity for RyR2 reduces the inhibition of RyR2 by Ca^2+^/CaM, and is combined with an increase in CaMKIIδ phosphorylation activity, which promote the opening of the RyR2 channel. Both phenomena lead to an increase in Ca^2+^ release from the SR, promoting arrhythmia.

In summary, both CPVT-associated variants CaM-N53I and CaM-A102V affect CaM–RyR2 structure-function relationship, resulting in Ca^2+^ release from the SR, via unique molecular mechanisms. Based on our findings, we propose that CaM-N53I likely acts through a loss of direct inhibition of RyR2 (subtle alterations in local structures), whereas for the CaM-A102V mutant, there is a finely tuned balance between loss of direct inhibition of RyR2 (higher *K*_d_) and increased channel activation via CaMKIIδ phosphorylation (Fig. 7). The loss of inhibition and overactivation of RyR2 triggers abnormal Ca^2+^ release from the SR. The subsequent increase in cytoplasmic Ca^2+^ concentration would then promote anomalous cardiac muscle contractions and generate irregular heartbeats, which are characteristic of CPVT syndrome. Interestingly, our data demonstrate a potential role for CaMKIIδ in the molecular aetiology of the disease, which could open up new therapeutic avenues.

## MATERIALS AND METHODS

### Plasmids

For recombinant protein expression, the human CaM gene was subcloned from the pGEX-6P-1 vector (Lian et al., 2014) into pE-SUMOPro by restriction-ligation (BsaI/XbaI) using 5′-CAGGTCTCAAGGTATGGCTGACCAACTGACTG-3′ and 5′-CATCTAGATTATCACTTTGCTGTCATCATTTG-3′. A series of site-directed mutagenesis reactions were performed, following the QuikChange protocol (Agilent Technologies), to generate CPVT-associated CaM constructs. The primers used were as follows: N53I, 5′-AGCAGAGTTACAGGACATGATTATTGAAGTAGATGCTGATG-3′; and A102V, 5′-GCAATGGCTATATTAGTGTTGCAGAACTTCGCCATGT-3′.

For the Ca^2+^ imaging experiments, CaM genes were subcloned from pE-SUMOPro into pHIV-dTomato (Addgene plasmid, 21374) vectors by Gibson Assembly (NEBuilder) following the manufacturer's recommendations and the NEBuilder assembly tool website. In these constructs, the IRES sequence allows CaM and dTomato to be co-expressed under the control of the same promoter as two distinct proteins.

All resulting vectors were confirmed by DNA sequencing (DNA Sequencing and Services, University of Dundee, UK). The molecular construct for expressing the human RyR2 (pcDNA-3/eGFP-hRyR2) was a kind gift from Dr Chris George (Swansea University Medical School, UK). pHIV-dTomato was a gift from Bryan Welm.

### Peptides

To circumvent the technical challenges associated with the production and use of full-length RyR2, we used two synthetic peptides (short and long) encompassing the CaMBD for our biophysical experiments: RyR2_3583-3603_ (KKAVWHKLLSKQRKRAVVACF) and RyR2_3581-3608_ (RSKKAVWHKLLSKQRKRAVVACFRMAP). It has been shown that the deletion of residues 3583-3603 abolishes the interaction between CaM and RyR2 (Tian et al., 2013; Yamaguchi et al., 2003), suggesting that this domain is essential for CaM binding. In addition, a recent cryo-EM structure of CaM in complex with RyR2 confirmed that residues 3593-3607 in the central domain represents the primary interface with CaM (Gong et al., 2019). The RyR2 peptides were synthesised and high-performance liquid chromatography-purified by GenicBio (China), and purity was estimated as >95% through mass spectrometry.

### Expression and purification of CaM proteins

Overexpression of CaM protein variants was performed in *Escherichia coli* BL21(DE3) STAR. Cells were grown in 1 l of 2× YT medium (16 g l^−1^ tryptone, 10 g l^−1^ yeast extract and 5 g l^−1^ NaCl) at 37°C while shaken at 200 rpm. Expression was induced by 0.5 mM isopropyl β-D-1-thiogalactopyranoside when OD_600nm_ reached 0.5-0.8, for 16-20 h at 18°C. Cells were resuspended in 50 mM Na^+^-HEPES (pH 7.5) and 200 mM NaCl containing Proteoloc protease inhibitor cocktail (Expedeon). Lysis was performed by incubation with 1 mg ml^−1^ lysozyme (Biozyme) on ice for 30 min followed by sonication (VibraCell, Jencons PLS). Lysates were then incubated with 500 U of BaseMuncher endonuclease (Expedeon) to hydrolyse RNA and DNA contaminants, and clarified through ultracentrifugation at 100,000 ***g***. Protein-containing supernatant was purified on an AKTA Start (GE Healthcare) using a 5 ml HisTrap HP column (GE Healthcare) equilibrated with 50 mM Na^+^-HEPES (pH 7.5) and 200 mM NaCl. Bound proteins were eluted by a linear gradient of 0-500 mM imidazole. The His-SUMO tag was cleaved from the protein by SUMO-protease added to a ratio of 2000:1 (protein-protease), for 1 h at 30°C. The untagged CaM proteins were further purified on a 5 ml HisTrap HP and recovered in the flow through. The final step of purification was performed on an AKTA Pure (GE Healthcare) equipped with a Superdex 75 (16/600) size exclusion column (GE Healthcare) equilibrated with 20 mM Na^+^-HEPES (pH 7.5) and 50 mM NaCl.

For NMR measurements, *E. coli* BL21(DE3) STAR cultures producing uniformly isotopic-labelled protein were grown in minimal medium containing 88 mM Na_2_HPO_4_, 55 mM KH_2_PO_4_, 30 μM thiamine-HCl, 136 μM CaCl_2_·2H_2_O, 1 mM MgSO_4_·7H_2_O, 19 mM ^15^NH_4_Cl and 22 mM glucose (or ^13^C glucose for triple resonance NMR experiments). Overexpression and purification were performed as described above. Isotopically labelled reagents were purchased from Cambridge Isotope Laboratories.

Unlabelled and isotopically labelled protein purity was determined by SDS-PAGE (NuPAGE 4-12% Bis-Tris, Life Technologies), and relevant fractions were concentrated using an Amicon Ultra-15 centrifugation unit (Millipore) with a 3 kDa cutoff. Purified proteins were aliquoted, flash-frozen in liquid nitrogen and stored at −80°C.

### Protein concentration measurements

Protein and peptide concentrations were measured by spectrophotometry at 280 nm (Nanodrop, Thermo Scientific) using ε_0_ (CaM)=2980 M^−1^.cm^−1^, ε_0_ (SUMO-protease)=1490 M^−1^.cm^−1^ and ε_0_ (RyR2 peptides)=5500 M^−1^.cm^−1^. Molar extinction coefficients were calculated from the amino acid composition using the ExPASy/ProtParam program (Gasteiger et al., 2005).

### Isothermal titration calorimetry

Experiments were performed in assay buffer [50 mM Na^+^-HEPES, 100 mM KCl and 2 mM MgCl_2_ (pH 7.5)] supplemented with either 5 mM CaCl_2_ or 5 mM EGTA for Ca^2+^-dependent or -independent binding events, respectively. EGTA was prepared as a stock solution (0.5 M with pH adjusted to pH 7.5 with KOH) and then added to the assay buffer (5 mM final) to prevent any final pH changes.

RyR2_3583-3603_ and RyR2_3581-3608_ peptides were titrated against CaM proteins across 20 injections (2 μl each) lasting 4 s with a 180-s grace period inbetween. Peptide was typically titrated into the cell at a tenfold higher concentration than CaM. CaM concentrations used were 10-45 µM in CaCl_2_ and 140-180 µM in EGTA.

All titrations were performed using MicroCal iTC200 and automated PEAQ-ITC systems (Malvern) at 25°C with continuous stirring at 800 rpm. Data were processed using MicroCal PEAQ-ITC software and fitted according to a one-site binding model.

### Secondary structure determination of CaM proteins

Circular dichroism spectra were recorded using a JASCO J-1100 spectrometer equipped with a JASCO MCB-100 mini circulation bath for temperature control. Far-UV circular dichroism spectra (180-260 nm) were recorded at 20°C in a 0.1 cm path length quartz cell (5 spectra accumulations, scan rate 50 nm min^−1^). Proteins (5 µM) were measured in 1 mM CaCl_2_ or 1 mM EGTA. Secondary structure content was determined using the CDSSTR prediction algorithm (DichroWeb online server, reference set3) (Johnson, 1999; Whitmore and Wallace, 2004, 2008).

### Protein susceptibility to protease degradation

Purified CaM (5 μM) was incubated with increasing concentrations of AspN endoproteinase (New England Biolabs) ranging from 0 to 300 µg ml^−1^ diluted in AspN-reaction buffer consisting of 50 mM Tris-HCl (pH 8.0) and 2.5 mM ZnSO_4_. Enzymatic reactions were incubated at 37°C for 30 min either in the presence of 5 mM CaCl_2_ or 5 mM EGTA, ±RyR2_3583-3603_ peptide (12 µM). The amount of intact protein was assessed by SDS-PAGE (NuPAGE 4–12% Bis-Tris, Life Technologies) and Coomassie staining (InstantBlue, Thermo Fisher Scientific). Images were taken using a ChemiDoc XRS+ transilluminator (Bio-Rad) and quantified by densitometry using Fiji software (Schindelin et al., 2012).

### Protein thermal stability

Thermal stability of CaM proteins was determined by following the unfolding of the α-helices at 222 nm using circular dichroism (JASCO J-1100 circular dichroism spectrometer equipped with a JASCO MCB-100 mini circulation bath for temperature control, 200 µL quartz cuvette, 0.1 cm path length). Data were collected in 2 mM Na^+^-HEPES buffer containing 1 mM EGTA and 10 µM CaM, from 15 to 80°C using 300 s equilibration time, 1°C increment (1°C min^−1^) and three accumulations. Signal recorded at each temperature was normalised as a fraction of total signal change and subjected to Boltzmann sigmoid analysis to obtain melting point (T_m_).

### NMR spectroscopy

All NMR spectra were collected at 30°C (303K) on an Avance III 800 MHz spectrometer equipped with [^1^H, ^15^N, ^13^C]-cryoprobes (Bruker)*.* ^1^H-^15^N HSQC experiments consisted of titrations of ^15^N-labelled CaM variants (100 µM) with unlabelled RyR2 peptide. Peptide titrations were performed in 20 mM Na^+^-HEPES (pH 7.5), 50 mM NaCl, 1 mM CaCl_2_ and 10% (v/v) D_2_O, with the stepwise addition of RyR2_3583-3603_ peptide to the protein sample after each recording to achieve 0.5:1, 1:1, 2:1 and 4:1 peptide:protein molar ratio. For chemical shift perturbation analysis, raw data were processed using Bruker TopSpin software. Resonance peaks were analysed and assigned using CcpNmr software (Vranken et al., 2005).

Backbone amino acid assignments for CaM variants were transferred from previously published solution NMR structures (Biological Magnetic Resonance Data Bank, 6541; Kainosho et al., 2006). Ambiguous peaks were assigned using standard triple resonance experiments CBCA(CO)NH and CBCANH obtained using 1 mM ^13^C-^15^N CaM-WT. Chemical shift differences were expressed in ppm as Δδ=[(ΔH)^2^+(0.15ΔN)^2^]^1/2^. Residues with chemical shifts equal to or less than 0.03 ppm were deemed non-movers, the remaining shifts were categorised into colour by increasing margins of 0.03 ppm and mapped onto the structure of Ca^2+^/CaM using PyMOL to illustrate a surface representation of the chemical shift derivations.

### X-ray crystallography

Crystals for Ca^2+^/CaM-WT–RyR2_3583-3603_, Ca^2+^/CaM-N53I–RyR2_3583-3603_ and Ca^2+^/CaM-A102V–RyR2_3583-3603_ proteins were grown at 20°C using the sitting drop vapour diffusion method (1 mM CaM, 1 mM CaCl_2_ and 1.2 mM RyR2_3583-3603_ peptide). Ca^2+^/CaM-WT–RyR2_3583-3603_ crystallised in 0.1 M sodium acetate (pH 4.5), 0.2 M ammonium acetate and 10% (w/v) polyethylene glycol (PEG) 4000. Ca^2+^/CaM-N53I–RyR2_3583-3603_ crystallised in 0.1 M sodium acetate trihydrate (pH 4.5) and 10% (w/v) PEG 10,000. Ca^2+^/CaM-A102V–RyR2_3583-3603_ crystallised in 0.1 M sodium acetate trihydrate (pH 4.0) and 25% (w/v) PEG 1500.

Crystals were cryoprotected using 20% (w/v) PEG 400, and diffraction data were collected using the Diamond synchrotron beamline I03 (CaM-N53I and CaM-A102V) and SOLEIL Proxima 1 (CaM-WT). Data were processed by xia2/DIALS (Winter et al., 2018) and scaled by AIMLESS (Evans, 2006). The structure for WT protein was solved by molecular replacement with MOLREP (Vagin and Teplyakov, 2010), using Ca^2+^/CaM-WT–RyR1_3614-3643_ (PDB, 2BCX) as a search model (Maximciuc et al., 2006). The structures of variants were refined starting from the WT structure using REFMAC5 (Murshudov et al., 1997) in the CCP4 program suite (Potterton et al., 2003; Winn et al., 2011). The final steps of refinement for Ca^2+^/CaM-WT–RyR2_3583-3603_ were performed with anisotropic B-factors and hydrogens. Rebuilding of the model between refinement cycles and adding water molecules was performed in COOT (Emsley and Cowtan, 2004). The quality of the models was assessed on the MolProbity server (Chen et al., 2010).

A summary of diffraction data, refinement statistics and the quality indicators for the structures are featured in Table S2. PDB codes are 6XXF (Ca^2+^/CaM-WT–RyR2_3583-3603_), 6XY3 (Ca^2+^/CaM-N53I–RyR2_3583-3603_) and 6XXX (Ca^2+^/CaM-A102V–RyR2_3583-3603_).

### CaMKIIδ phosphorylation activity measurements

Six-week-old male CD1 mice (Charles River) were humanely killed by cervical dislocation (schedule 1 procedure) in accordance with the Animals (Scientific Procedures) Act (1986) under Establishment Licence 40/2408 and with approval by the University of Liverpool Animal Welfare Committee and Ethical Review Body. Mice hearts were surgically removed and homogenised with a blender in 3 ml of extraction buffer [20 mM Tris (pH 8.0), 2 mM EDTA, 2 mM EGTA, 2 mM dithiothreitol (DTT) and complete protease inhibitor ULTRA mini]. Heart lysates were clarified by centrifugation and used as a CaMKIIδ source for the assay. CaMKIIδ phosphorylation activity was determined using [γ^32^P] ATP (PerkinElmer) and the SignaTECT Calcium/Calmodulin-Dependent Protein Kinase Assay System (Promega) according to the manufacturer's recommendations. Purified CaM and CPVT-associated mutant recombinant proteins were added to the reaction mix (1 µM) to determine the effect of disease-associated CaM variants on CaMKIIδ phosphorylation activity. Scintillation levels were measured using a Tri-Carb 2910 TR low activity liquid scintillation analyser (PerkinElmer).

### Time course of CaMKIIδ autophosphorylation

GST-CaMKIIδ (300 nM, Abcam, ab84552) and 1 µM calmodulin were incubated in 50 mM K^+^-HEPES (pH 7.5), 100 mM KCl, 2 mM MgCl_2_, 5 mM DTT and 100 µM CaCl_2_ at room temperature. The reaction was started by adding 300 µM ATP and was terminated using SDS-containing Laemmli sample buffer at predefined timepoints (0 s, 15 s, 30 s, 60 s, 120 s and 300 s). Post separation of proteins by SDS-PAGE (NuPage 4-12% Bis-Tris), proteins were electrotransferred from the gel to a nitrocellulose membrane using an iBlot 2 gel transfer device (7-min protocol consisting of three steps: 20V for 1 min, 23V for 4 min and 25V for 2 min). The membranes were blocked with 5% (w/v) fat-free powdered milk in T-TBS buffer [0.1% (v/v) Tween 20, 50 mM Tris-HCl (pH 7.6) and 150 mM NaCl]. Membranes were then probed overnight at 4°C with mouse anti-GST (Sigma-Aldrich, G1160) and rabbit anti-phospho T287 (Abcam, ab182647) monoclonal primary antibodies at 1/1000 and 1/500 dilutions, respectively. Next, membranes were washed in T-TBS and incubated for 1 h at room temperature with IRDye 680RD donkey anti-mouse (LI-COR, 926-68072) and IRDye 800CW donkey anti-rabbit (LI-COR, 926-32213) IgG secondary antibodies at a 1/10,000 dilution. The bands were visualized using an Odyssey CLx infrared imaging system, and the intensity of the bands were quantified by densitometry using Fiji.

### Cell culture, transfection and Ca^2+^ imaging

HEK293T cells (1×10^5^, American Type Culture Collection, UK) were seeded onto 35 mm poly-lysine-treated glass-bottomed dishes (MatTek Corporation). Cells were co-transfected with eGFP-hRyR2 (pcDNA3) and CaM variant (dTomato) plasmids in an equal molar ratio using Effectene reagent (Qiagen) according to the manufacturer's instructions. Cells were imaged after 48 h of expression and loading with 10 µM Calbryte 520 AM (AAT Bioquest). Imaging was carried out at 37°C/5%CO_2_ (OKO lab incubation chamber) using a 3i Marianas spinning-disk confocal microscope equipped with a Zeiss AxioObserver Z1, a 20×/0.8 Plan-Apochromat air objective and a 3i Laserstack as an excitation light source (488nm, for Calbryte/eGFP; 561nm, for dTomato). Emitted light was collected through single bandpass filters (CSU–X filter wheel, Yokogawa, Tokyo, Japan) onto a complementary metal-oxide semiconductor (CMOS) camera (Orca Flash 4.0, Hamamatsu, Japan).

Cells were covered with Krebs buffer [200 µl (pH 7.4), containing 1.3 mM CaCl_2_] and data were acquired at a rate of ∼5 frames/s using SlideBook v.6 software at 1024×1024 pixel resolution. Spontaneous Ca^2+^ oscillations and ER load (10 mM caffeine) were recorded from cells co-expressing RyR2 (eGFP) and CaM variants (dTomato). Non-RyR2-associated Ca^2+^ response was obtained after the addition of 10 µM carbachol or 2 µM thapsigargin from cells expressing CaM variants (dTomato). Calbryte 520 fluorescence signals were measured from regions of interest outlining individual cells using Ilastik for segmentation and Fiji for the intensity recording within the segmented areas (Berg et al., 2019; Schindelin et al., 2012). Kinetic parameters of Ca^2+^ release were quantified using Analyse Spikes applet for MatLab (Dr Aled Jones, Queen Mary University of London, UK).

For intracellular Ca^2+^ measurements, HEK293T cells expressing CaM variants (dTomato) were loaded with 5 µM fura-2 AM for 1 h at 37°C. Cells were imaged using a Nikon Eclipse TE200 microscope equipped with a 20×/0.45 Plan Fluor air objective, 48 h post transfection. Cells were illuminated at 340 and 380 nm using a PTI monochromator (PTI, Birmingham, NJ, USA) and fluorescence emissions were captured above 520 nm using an Andor Zyla 4.2 sCMOS camera (Andor Technology, Belfast, UK). Images were acquired using Winfluor 4.0 software (University of Strathclyde). Data were processed using Fiji (Schindelin et al., 2012) and expressed as the ratio of the 340:380 nm signals.

### Data analysis and statistics

Experiments were performed at least in triplicates and analysed using GraphPad Prism. Significance level was obtained using a two-tailed unpaired Student's *t*-test, one-way ANOVA or two-way ANOVA. *P*-values are represented by stars (*P<0.05, **P<0.01, ***P<0.001 and ****P<0.0001). Structure representations were created using the PyMOL Molecular Graphics System software (v2.0.7) and figures were generated using CorelDRAW 2019.

## Supplementary Material

Supplementary information

Reviewer comments
