# Supplementary material for: CPVT-associated calmodulin variants N53I and A102V dysregulate Ca2+ signalling via different mechanisms
Source: J Cell Sci. 2022 Jan 18;135(2):jcs258796. doi: 10.1242/jcs.258796 (PMC8917356; doi:10.1242/jcs.258796)
Supplement: Supplementary information [file joces-135-258796-s1.pdf]

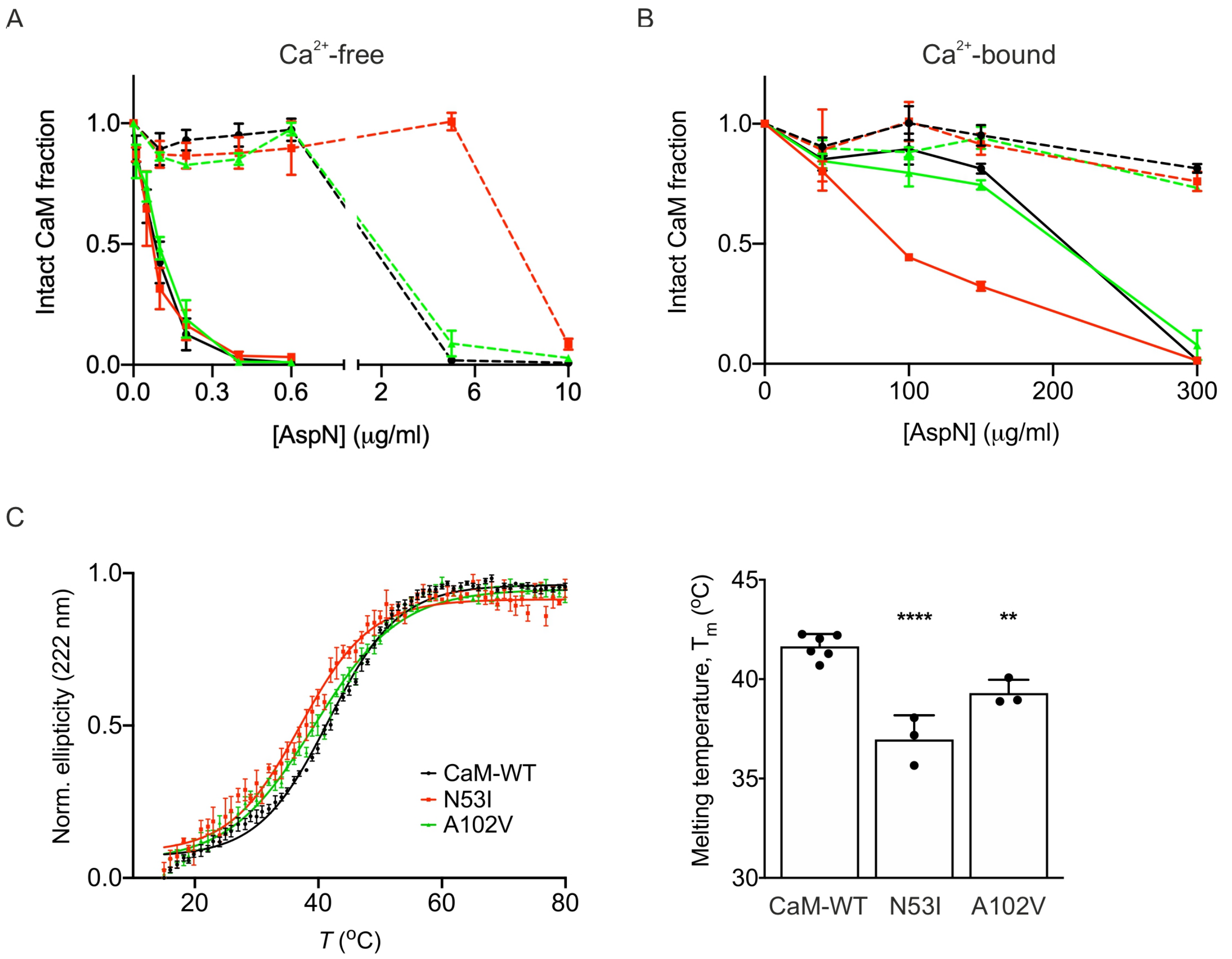

Fig. S1. Arrhythmogenic mutations N53I and A102V affect protease and thermal stability of CaM. (A-B) AspN limited proteolysis of CaM-WT and mutants in (A) absence and (B) presence of Ca^{2+}. Purified CaM proteins (CaM-WT, black; CaM-N53I, red; CaM-A102V, green) were mixed with increasing [AspN] for 30 minutes at 37 °C, with (dotted line) or without (plain lines) pre-incubation with $RyR2_{3583-3603}$ peptide. The fraction of intact CaM was determined by SDS-PAGE and Coomassie staining. Bands were quantified by densitometry analysis (Fiji). Data were normalised and expressed as mean ± SEM. Experiments were performed in 3-5 independent replicates.

Number of experimental replicates for apo-conditions was: n=4 for CaM-WT, n=4 for CaM-N53I, n=5 for CaM-A102V, n=4 for CaM-WT:RyR2, n=4 for CaM-N53I:RyR2 and n=4 for CaM-A102V:RyR2. Number of experimental replicates for Ca^{2+}-saturating conditions was: n=3 for CaM-WT, n=3 for CaM-N53I, n=3 for CaM-A102V, n=3 for CaM-WT:RyR2, n=3 for CaM-N53I:RyR2 and n=3 for CaM-A102V:RyR2.

(C) Thermal unfolding of CaM proteins (10 μM) monitored by circular dichroism recorded at 222 nm from 15 to 80 °C, in the presence of 1 mM EGTA. Data were normalised and expressed as mean ± SEM. Melting temperature (T_m) was obtained by fitting the traces to the Boltzmann equation. Number of experimental replicates was: n=6 for CaM-WT, n=3 for CaM-N53I and n=3 for CaM-A102V. Differences between three groups were determined using one-way ANOVA with the Dunnett's post-hoc test. **$P < 0.01$, ****$P < 0.0001$, vs. CaM-WT.

Journal of Cell Science • Supplementary information

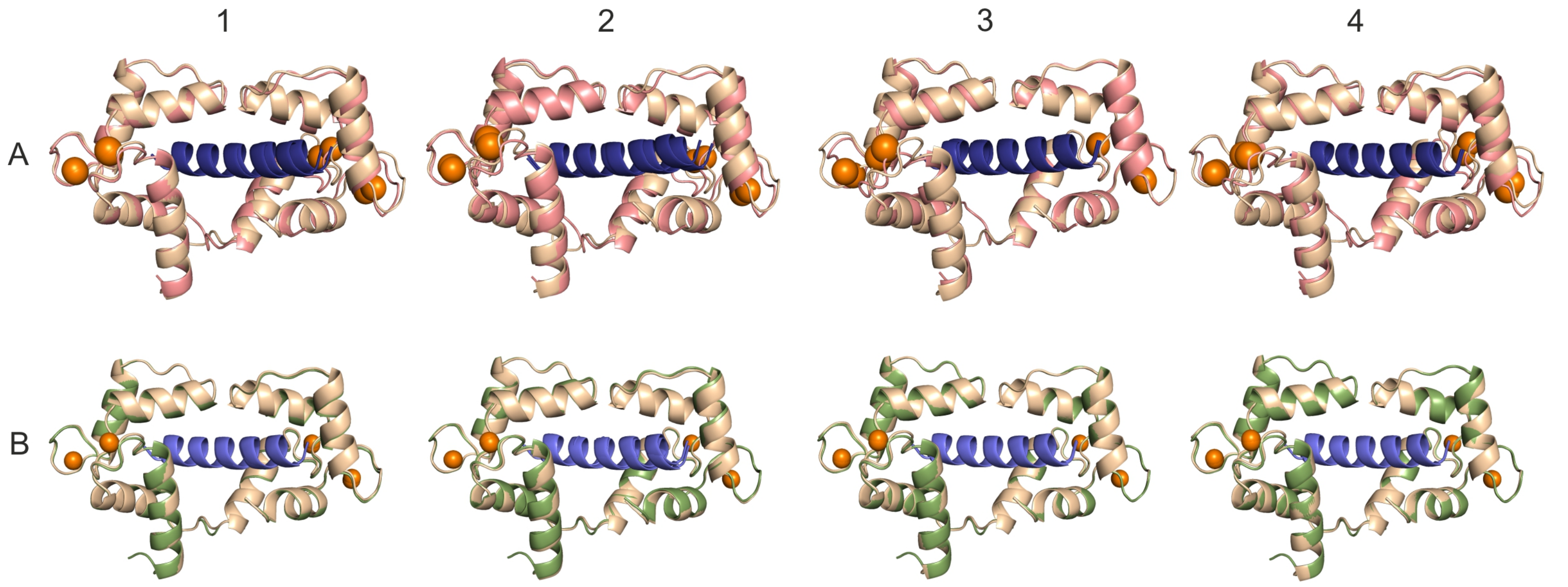

Fig. S2. Structural superposition of Cα atoms of Ca^{2+}/CaM-RyR peptide complexes. Superimposition of the crystal structure of Ca^{2+}/CaM-WT:RyR2$_{3583\text{-}3603}$ and (A) Ca^{2+}/CaM-N53I:RyR2$_{3583\text{-}3603}$ or (B) Ca^{2+}/CaM-A102V:RyR2$_{3583\text{-}3603}$. Superimposition was based on (1) N-terminal region (residues 5-64), (2) flexible helix region (residues 65-92), (3) C-terminal region (residues 93-148) or (4) RyR2 peptide.

CaM-WT is displayed in beige (CaM-WT from PDB 2BCX is displayed in yellow), CaM-N53I in salmon and CaM-A102V in green. RyR2$_{3583\text{-}3603}$ (KKAVWHKLLSKQRKRAVVACF) is shown in blue. Ca^{2+} ions are represented as orange spheres. Images were generated using PyMOL software.

Journal of Cell Science • Supplementary information

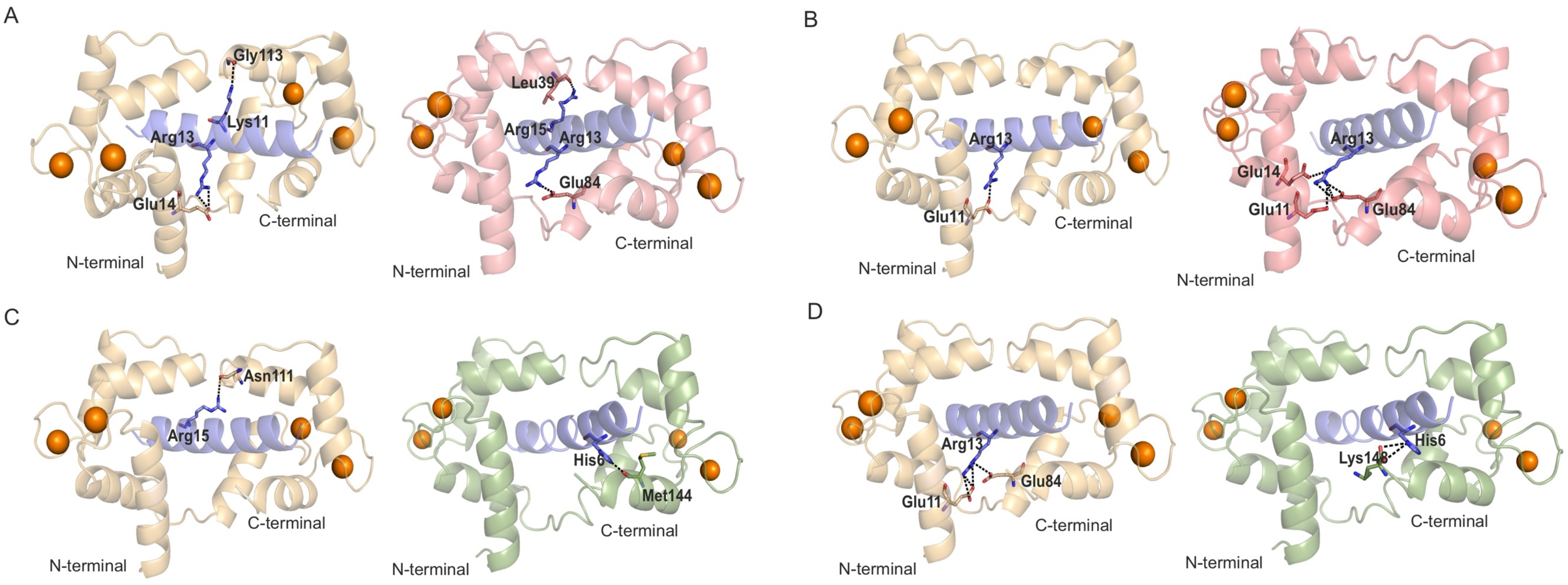

Fig. S3. Comparison of the crystal structures of Ca^{2+}/CaM-WT:RyR2$_{3583\text{-}3603}$ (PDB 6XXF), Ca^{2+}/CaM-N53I:RyR2 (PDB 6XY3) and Ca^{2+}/CaM-A102V:RyR2$_{3583\text{-}3603}$ (PDB 6XXX). Cartoon representation of (A-B) Ca^{2+}/CaM-WT:RyR2$_{3583\text{-}3603}$ (left; CaM in beige and RyR2$_{3583\text{-}3603}$ peptide in blue colour) and Ca^{2+}/CaM-N53I: RyR2$_{3583\text{-}3603}$ peptide complex (right; CaM-N53I in salmon and RyR2 peptide in blue colour). (C-D) Ca^{2+}/CaM-WT:RyR2$_{3583\text{-}3603}$ (left; CaM in beige and RyR2$_{3583\text{-}3603}$ peptide in blue colour) and Ca^{2+}/CaM-A102V: RyR2$_{3583\text{-}3603}$ peptide complex (right; CaM-A102V in green and RyR2 peptide in blue colour).

Residues from Ca^{2+}/CaM and corresponding peptides involved in (A, C) H-bonds and (B, D) salt bridges unique to each are represented in sticks with interactions marked in black dashed lines. Ca^{2+} ions are represented as orange spheres.

Peptide residues are numbered based on the SI index in Table S4.

RyR2$_{3583\text{-}3603}$ (KKAVWHKLLSKQRKRAVVACF) is numbered 1-21. Images were generated using PyMOL software.

Journal of Cell Science • Supplementary information

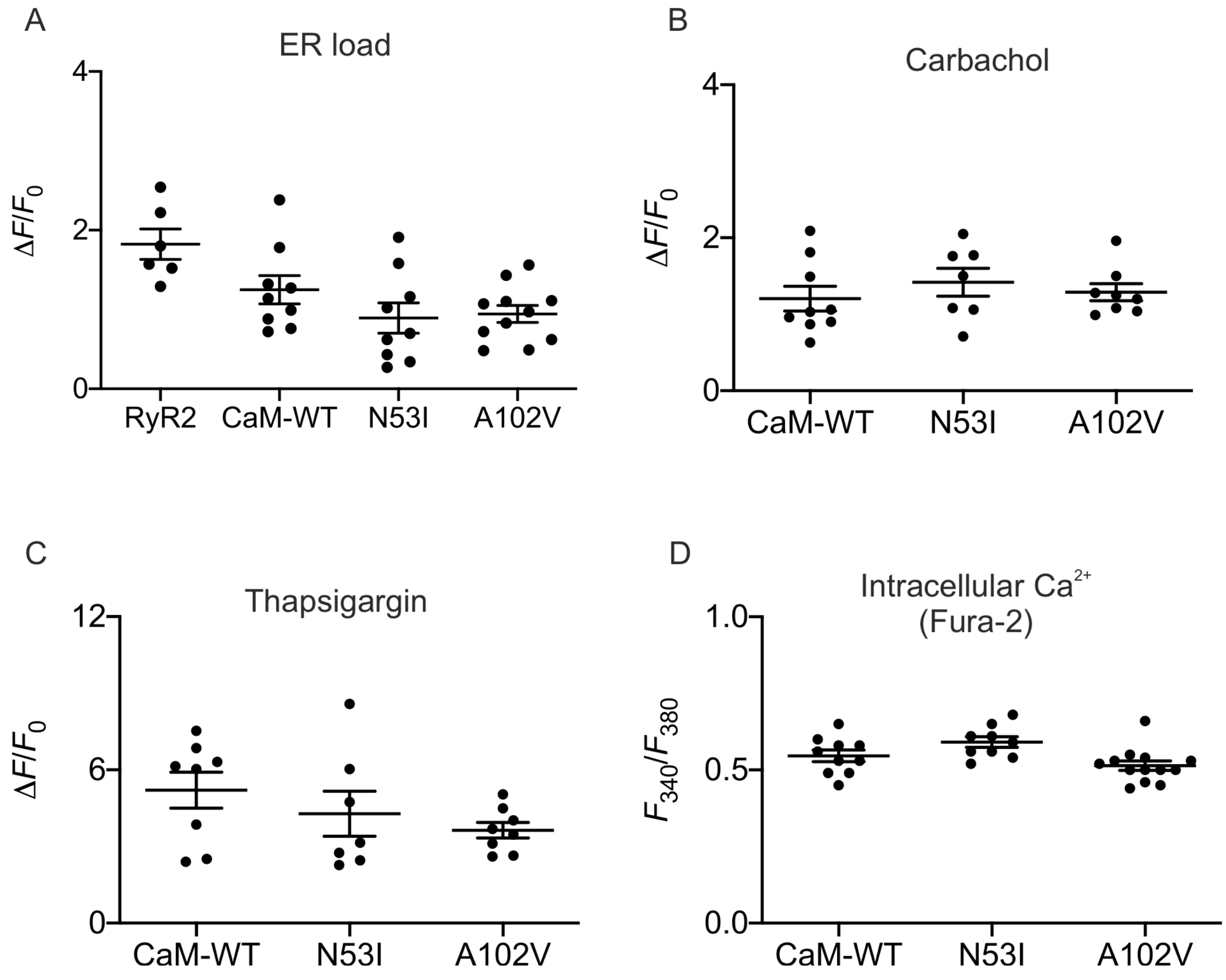

Fig. S4. Over-expression of CaM-WT and CPVT-associated CaM variants did not affect ER-load (caffeine), non-RyR Ca^{2+} response (carbachol, thapsigargin) and intracellular Ca^{2+} concentrations.

(A-C) HEK293T cells transfected with CaM variants ± RyR2 were loaded with Calbryte 520 to monitor intracellular Ca^{2+} concentration changes. Live cells were imaged on a 3i spinning-disk confocal microscope after stimulation with (A) caffeine, (B) carbachol or (C) thapsigargin. (D) HEK293T cells transfected with CaM variants were loaded with Fura-2 to monitor intracellular Ca^{2+} concentration. Live cells were analysed using a Nikon Eclipse epifluorescence microscope and ratiometric imaging.

Data were processed using Fiji and expressed as mean ± SEM. Number of experimental replicates for the ER load experiments (N=dishes) was: N=6 for RyR2, N=9 for RyR2 + CaM-WT, N=9 for RyR2 + CaM-N53I and N=11 for RyR2 + CaM-A102V. Number of experimental replicates for the carbachol experiments (N=dishes) was: N=9 for CaM-WT, N=7 for CaM-N53I and N=8 for CaM-A102V. Number of experimental replicates for the thapsigargin experiments (N=dishes) was: N=8 for CaM-WT, N=7 for CaM-N53I and N=8 for CaM-A102V. Number of experimental replicates for the intracellular Ca^{2+} experiments (N=dishes, n = fields of view) was: N=4, n=10 for CaM-WT; N=3, n=9 for CaM-N53I and N=6, n=13 for CaM-A102V. Differences between groups were determined using one-way ANOVA vs. CaM-WT.

Journal of Cell Science • Supplementary information

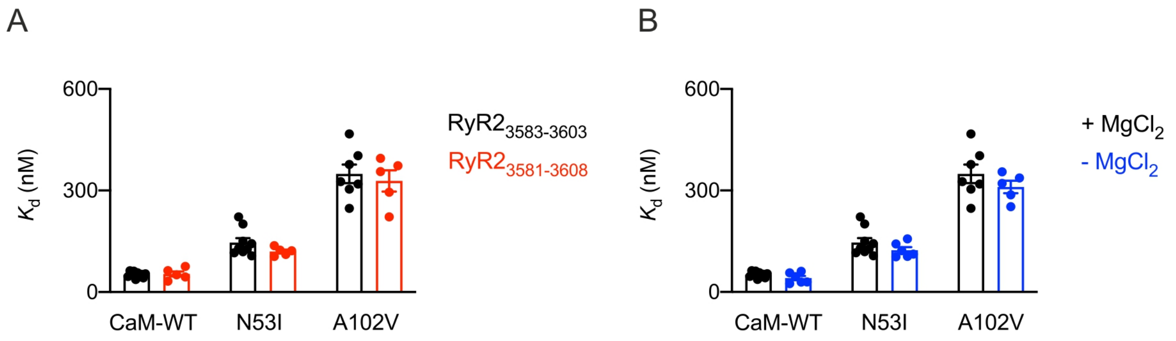

Fig. S5. Effect of peptide length and magnesium concentration on Ca^{2+}/CaM binding to RyR2 using ITC. (A) Affinity of the binding of Ca^{2+}/CaM variants to $RyR2_{3583\text{-}3603}$ (short) and $RyR2_{3581\text{-}3608}$ (long) obtained by fitting to one-site binding model. (B) Affinity of the binding of Ca^{2+}/CaM variants to $RyR2_{3583\text{-}3603}$ in the presence or absence of 2 mM $MgCl_2$ obtained by fitting to one-site binding model Data were processed using the MicroCal PEAQ-ITC software and expressed as mean ± SEM. Experiments were performed at least in 5 replicates, in the presence of 5 mM $CaCl_2$ at 25 °C.

Journal of Cell Science • Supplementary information

Table S1. The C-lobe of Ca^{2+}/CaM binds first to RyR2. Percentage of residue resonances assigned in the N and C lobes of various ^{15}N-labelled CaM variants from HQSC spectra analysis. Using 0.3 ppm as a threshold, the percentage of assigned resonances which have not significantly shifted in the presence of one molar equivalent $RyR2_{3583\text{-}3603}$ peptide are displayed for each CaM lobe (left-hand side). Percentage of assigned resonances which have not significantly shifted upon the addition of $RyR2_{3583\text{-}3603}$ peptide from one to two molar equivalents (right-hand side).

**CaM-WT**	**Unbound – 1.RyR2**		**1.RyR2 – 2.RyR2**	
	% peak assigned	% non-movers	% peak assigned	% non-movers
N-lobe	59.2	57.1	50.7	58.3
C-lobe	36.4	29.2	36.4	91.7

**N53I**	**Unbound – 1.RyR2**		**1.RyR2 – 2.RyR2**	
	% peak assigned	% non-movers	% peak assigned	% non-movers
N-lobe	45.1	43.8	42.3	56.7
C-lobe	34.9	26.1	33.3	81.8

**A102V**	**Unbound – 1.RyR2**		**1.RyR2 – 2.RyR2**	
	% peak assigned	% non-movers	% peak assigned	% non-movers
N-lobe	60.6	46.5	49.3	54.3
C-lobe	33.3	18.2	31.8	76.2

Journal of Cell Science • Supplementary information

Table S2. Data collection and refinement statistics.
Values in brackets are for the last resolution shell.

	Ca^{2+}/CaM-WT:RyR2$_{3583-3603}$	Ca^{2+}/CaM-N53I:RyR2$_{3583-3603}$	Ca^{2+}/CaM-A102V:RyR2$_{3583-3603}$
**Data collection Wavelength (Å)**	0.97857	0.97624	0.97624
**Beamline**	Proxima 1	I03	I03
**Detector**	Pilatus	Pilatus	Pilatus
**Space group**	$P2_12_12_1$	$P2_12_12_1$	$P2_12_12_1$
**Unit-cell dimensions (*a,b,c*) (Å)**	39.92, 41.71, 85.99	39.01, 42.70, 89.36	39.97, 41.67, 85.75
**Resolution (Å)**	37.56-1.70 (1.70-1.73)	44.72-2.00 (2.00-2.05)	27.34 - 1.23 (1.25 - 1.23)
***Rmerge* %**	9.2 (57.2)	10.0 (36.8)	3.8 (81.6)
**I/σ (last shell)**	12.5 (6.2)	8.9 (3.8)	18.45 (1.15)
**Completeness (%)**	100.0 (98.7)	100.0 (100.0)	98.48 (84.19)
**Redundancy**	5.9 (5.7)	5.3 (5.4)	5.66 (2.65)
**Half-set correlation $CC_{1/2}$**	0.991 (0.870)	0.994 (0.908)	5.66 (2.65)
**No. of reflections**	16416	10591	41749
**R_{work}/R_{free}**	18.0/20.5	20.8/25.9	13.7/19.1
**No. of atoms**			
Protein	1312	1295	1386
Ca ions	4	4	4
Water	140	50	219
**B factor ($Å^2$)**			
Protein	22.63	35.81	24.42
Ca ions	15.87	29.55	19.16
Waters	28.21	32.97	22.45
**R.M.S deviations**			
Bond length (Å)	0.013	0.008	0.017
Bond angles (°)	1.805	1.457	2.106
**PDB code**	**6XXF**	**6XY3**	**6XXX**

Journal of Cell Science • Supplementary information

Table S3. Structural RMSD between various regions of the Ca^{2+}/CaM-WT:RyR2$_{3583\text{-}3603}$ peptide complex and the Ca^{2+}/CaM-N53I:RyR2$_{3583\text{-}3603}$, Ca^{2+}/CaM-A102V:RyR2$_{3583\text{-}3603}$ peptide complex crystal structures.

RMSD values were obtained using PyMOL "super" command.

Ca^{2+}/CaM-WT:RyR2$_{3583\text{-}3603}$	Ca^{2+}/CaM-N53I: RyR2$_{3583\text{-}3603}$ RMSD (Å)	Ca^{2+}/CaM-A102V: RyR2$_{3583\text{-}3603}$ RMSD (Å)
Full complex	1.31	0.63
N-terminal region (residue 6 - 65)	0.90	0.72
Flexible helix (residue 66 - 93)	1.43	0.77
C-terminal region (residue 94 - 149)	0.77	0.52
RyR2$_{3583\text{-}3603}$ peptide	1.13	0.21

Table S4. Residues involved in H-bond and salt bridge interactions between RyR2$_{3583\text{-}3603}$ peptide and Ca^{2+}/CaM-WT, Ca^{2+}/ CaM-N53I and Ca^{2+}/CaM-A102V. Data were obtained using PDBePISA server.

		H-bond interactions			Salt bridge interactions		
SI.	RyR2$_{3583\text{-}3603}$	Ca^{2+}/CaM-WT	Ca^{2+}/CaM-N53I	Ca^{2+}/CaM-A102V	Ca^{2+}/CaM-WT	Ca^{2+}/CaM-N53I	Ca^{2+}/CaM-A102V
1	Lys3583	Glu123; Glu127	Glu127[1]	Glu123; Glu127	Glu123; Glu127	Glu127[1]	Glu123; Glu127
2	Lys3584	Glu127	Glu127	Glu127	-	-	-
3	Ala3585	-	-	-	-	-	-
4	Val3586	-	-	-	-	-	-
5	Trp3587	Met124	Met124	Met124	-	-	-
6	His3588	-	-	Met144	-	-	Lys148
7	Lys3589	Glu114	Glu114	Glu114	Glu114	Glu114	Glu114
8	Leu3590	-	-	-	-	-	-
9	Leu3591	-	-	-	-	-	-
10	Ser3592	-	-	-	-	-	-
11	Lys3593	Leu112; Gly113; Glu114	Leu112; Glu114	Leu112; Gly113; Glu114	Glu114	Glu114	Glu114
12	Gln3594	-	-	-	-	-	-
13	Arg3595	Glu11; Glu14	Glu11; Glu84	Glu11; Glu14	Glu11; Glu14; Glu84	Glu11; Glu14; Glu84	Glu14; Glu84
14	Lys3596	-	-	-	-	-	-
15	Arg3597	Asn111	Leu39; Asn111	-	-	-	-
16	Ala3598	-	-	-	-	-	-
17	Val3699	-	-	-	-	-	-
18	Val3600	-	-	-	-	-	-
19	Ala3601	-	-	-	-	-	-
20	Cys3602	Lys75	-[2]	Lys75	-	-	-
21	Phe3603	-	-	-	-	-	-

[1] Glu123 side-chain atoms are not resolved in the structure

[2] Lys75 side-chain atoms are not resolved in the structur

Journal of Cell Science • Supplementary information
