# Supplementary material for: CPVT-associated calmodulin variants N53I and A102V dysregulate Ca2+ signalling via different mechanisms
Source: J Cell Sci. 2022 Jan 18;135(2):jcs258796. doi: 10.1242/jcs.258796 (PMC8917356; doi:10.1242/jcs.258796)
Supplement: Reviewer comments [file joces-135-258796_review_history.pdf]

Journal of Cell Science

# CPVT-associated calmodulin variants N53I and A102V dysregulate $Ca^{2+}$ signalling via different mechanisms

Ohm Prakash, Marie Held, Liam F. McCormick, Nitika Gupta, Lu-Yun Lian, Svetlana Antonyuk, Lee P. Haynes, N. Lowri Thomas and Nordine Helassa
DOI: 10.1242/jcs.258796

**Editor**: John Heath

**Review timeline**
Original submission: 15 April 2021
Editorial decision: 22 July 2021
First revision received: 1 November 2021
Accepted: 29 November 2021

---

**Original submission**

First decision letter

MS ID#: JOCES/2021/258796

MS TITLE: Catecholaminergic polymorphic ventricular tachycardia (CPVT)-associated calmodulin variants N53I and A102V dysregulate calcium signalling via different mechanisms

AUTHORS: Ohm Prakash, Marie Held, Liam F McCormick, Lu-Yun Lian, Svetlana Antonyuk, Lee P Haynes, Nia Lowri Thomas, and Nordine Helassa
ARTICLE TYPE: Research Article

We have now reached a decision on the above manuscript.

To see the reviewers' reports and a copy of this decision letter, please go to: https://submit-jcs.biologists.org and click on the 'Manuscripts with Decisions' queue in the Author Area. (Corresponding author only has access to reviews.)

As you will see, the reviewers raise a number of substantial criticisms that prevent me from accepting the paper at this stage. They suggest, however, that a revised version might prove acceptable, if you can address their concerns. If you think that you can deal satisfactorily with the criticisms on revision, I would be pleased to see a revised manuscript. We would then return it to the reviewers.

*We are aware that you may be experiencing disruption to the normal running of your lab that makes experimental revisions challenging. If it would be helpful, we encourage you to contact us to discuss your revision in greater detail. Please send us a point-by-point response indicating where you are able to address concerns raised (either experimentally or by changes to the text) and where you will not be able to do so within the normal timeframe of a revision. We will then provide further guidance. Please also note that we are happy to extend revision timeframes as necessary.*

Please ensure that you clearly highlight all changes made in the revised manuscript. Please avoid using 'Tracked changes' in Word files as these are lost in PDF conversion.

© 2021. Published by The Company of Biologists under the terms of the Creative Commons Attribution License (https://creativecommons.org/licenses/by/4.0/).

I should be grateful if you would also provide a point-by-point response detailing how you have dealt with the points raised by the reviewers in the 'Response to Reviewers' box. Please attend to all of the reviewers' comments. If you do not agree with any of their criticisms or suggestions please explain clearly why this is so.

Reviewer 1

*Advance summary and potential significance to field*

The manuscript provides novel data on the effects of two CPVT mutations in calmodulin (CaM) on the structure CaM and the Ca2+/CaM:RyR23583-3603 complex.
Isothermal titration calorimetry, revealed that binding of both Ca2+/CaM-N53I and Ca2+/CaM-A102V to RyR23583-3603 is decreased by 3- and 7-fold respectively compared with WT CaM complex. High resolution crystal structures of the Ca2+/CaM:RyR23583-3603 complex indicated minor changes for the N53I variant while the A102V variant was similar to wild-type. Co-expression of either CaM-N53I or CaM-A102V with RyR2 in HEK293 cells significantly increased the duration of spontaneous Ca2+ transients relative to WT CAM, with CaM-A102V exhibiting a lower frequency of oscillations; observations indicative of disrupted CaM-mediated inhibition of RyR2. In addition CaMKIIδ phosphorylation activity was unchanged in N53I, but significantly increased in A102V. Together the results provide newstructural insight into molecular changes the allow N53I and A102V cause CPVT to reduce the normal inhibitory action of WT-CaM on RyR2. CaM-N53I reduces direct inhibition of RyR2 through an altered 3D structure; CaM-A102V decreases direct inhibition of RyR2 via weaker binding combined with an increased phosphorylation activity of CaMKIIδ. Significance for the field is the molecular insight and the potential for therapeutic development.

*Comments for the author*

Major points.
1. The amount of information the supplementary material was a distraction from the flow of reading as it required jumping back and forth between files.
This was particularly annoying as many of the main manuscript figures have very little information and a lot of unoccupied white space (Figures 2, 3, 4 5, 6 and 7, particularly later Figs). Most of the S figures could be included the main manuscript if the existing figures were rearranged and combined.
Similarly tables could be combined and included in the main manuscript.
2. P3. Information/references about the CaM A102V and CaM-N53I mutations previous studies: identification and human and model system studies. The two variants are named with no information apart from CPVT (without no reference).
Please include this information.
3. Discussion – how do you envisage the results could Enlarge on

Minor.
1. It is well known that EGTA is very acidic. Was the pH of the solution adjusted after addition of EGTA to the HEPES solution? Please clarify in the Results section on p.4 and in Methods.
2. P5, para 2, line 1 -typo “been” shound be “being”.
3. P8 line 4. Do you mean “previous studies” with the same variants?
4. P9 para 4. Provide a reference for the clincal phenotypes mentioned.

Reviewer 2

*Advance summary and potential significance to field*

This paper demonstrates that CPVT associated A102 calmodulin variant does not induce significant structural changes when bound to the RyR2 binding peptide at high Ca2+. This is similar to the subtle structural effect observed in the CPVT associated N53I calmodulin variant. These observations are interesting and leaves the field with the notion that RyR2 regulation is extremely sensitive to the slightest variation in the regulating calmodulin protein. The study demonstrates in contrast to N53I, the A102V calmodulin variant leads to increased CaMKIId phosphorylation. This

© 2021. Published by The Company of Biologists under the terms of the Creative Commons Attribution License (https://creativecommons.org/licenses/by/4.0/). 

activity is novel and demonstrates that there may be significant differences in the molecular pathogenic mechanisms for the rare individual calmodulin mutations, despite the occurrence of seemingly identical phenotypes.

*Comments for the author*

The authors determined high-resolution structures and binding (ITC) of two CPVT associated calmodulin mutations, N53I and A102V in complex with a short form of the calmodulin binding domain from RyR2. The two CaM variants are further characterized by CD (secondary structure and folding stability), and protease susceptibility. The data for A102V variant are new – similar data has been published for the N53I variant.
They further determine intracellular Ca2+ oscillation kinetics in HEK cells transfected with RyR2 and the CaM variants. Finally, the CaM variant effect on CaMKIId phosphorylation in murine heart extracts was determined.
There are a number of issues that the authors needs to adress.

Major concerns:
1. The choice of length of the RyR2-CaMBD peptide used in this study could be problematic: in this manuscript, CaMBD2 ends at amino acid 3603 (the Phenylalanine involved in binding the N-lobe of CaM, thus introducing a negative charge from the C-terminal carboxylic acid in immediate vicinity of the CaM binding). The published crystal structure of the CaM-RyR2(PDB 6Y4P) shows that the following three amino acids 3604-3607 (missing in this study) are quite close to parts of the N-lobe (amino acids 50, 51,54 and 71).
Moreover, as the authors state, in the manuscript, a cryo-EM structure by Gong et al (2019) also showed that amino acids up to and including 3607 form the interaction site with CaM. Thus, the premature truncation at amino acid 3603 seems not an obvious choice and the premature truncation might be the cause for the differences in some of the results of this study and a previous study (Holt 2020): the previous study did not find any difference in affinity of CaM for CAMBD2 in the presence of Ca between the wt and the N53I variant (using amino acids 3581-3611)– this study does find a factor 3 different Kd (using amino acids 3583-3603). The authors comment on this discrepancy and explain it with the presence of Mg ions in their ITC experiment. It is not straightforward to see how 2mM Mg (which is free in solution) can cause a factor 3 drop in Kd of an interaction not involving Mg, while it is much more likely that a missing stretch of amino acids in CAMBD2, some of which close to the mutation site in CaM, would influence the Kd. The authors needs to address this. One option would be to demonstrate that the difference in Kd is maintained with a longer CaMBD2 peptide. If MG causes the difference, this should be ease to demonstrate (perform ITC without Mg).
2. Paragraph entitled ”the interaction of CaM with RyR2 is driven by the C-lobe of CaM”: Please mention, whether this paragraph deals with the Ca-free or Ca-bound version of CaM? (The methods section suggests that Ca was present, but it would be nice to have this clearly stated here, too. The observed “two-step”
binding is very strange. If it were not for the ITC clearly yielding a 1:1 stoichiometry, those data would suggest a 2:1 stoichiometry. How would additional RyR2 free in solution impact on the structure of the already formed complex? With the given Kd values and concentrations, at 100 uM of both RyR2 and CaM, 98% of Cam should be bound to RyR. moreover, both N and C-lobe change a lot of chemical shifts upon binding in the first step up to 1:1 ratio. The fact, that more C-lobe resonances shift than N-lobe resonances, cannot be used to argue that the “C-lobe drives the interaction”. Whether or not an interaction leads to a chemical shift change, depends on the nature of the interaction and on nearby amino acids.
Nevertheless, the observed shift changes in the N-lobe upon continued addition of RyR2-CAMBD2 are not straightforward to understand.
3. P.8, end of 1st paragraph “”[..] indicating that only the N53I
variant induced significant structural changes.” This statement should be modified, since neither the results reported here not previous results by Holt et al show any indication of significant structural changes.
4. The abstract should thus also be modified to reflect point 3. The study does not provide evidence that the suggested subtle structural changes in N53I, when in complex with a truncated CaM binding peptide (which is contrasting the previously published by Holt et al) are the cause of RyR2 dysregulation.

© 2021. Published by The Company of Biologists under the terms of the Creative Commons Attribution License (https://creativecommons.org/licenses/by/4.0/).

5. The increased protease susceptibility of the N53I variant is more likely to come from an increase in local intramolecular dynamics, rather than a significant structural change. The authors should change the wording of their conclusions.
6. Page 8, 3rd paragraph: the two lobes of CaM are rather independent of each other and an intramolecular allosteric network between the two lobes would be really unusual. Moreover, the previous study by Holt et al did not report a significant shift change in any C-lobe amino acid upon mutating N53I.
7. Page 8, last paragraph "We deposited the first high-resolution structures…": in the strictest sense, the statement is correct, since no structure with the exact same amino acid range 3583-3603 has been published before. However, the statement is somewhat misleading, ignoring the structures presented by Holt et al. Modify appropriately.
8. Page 9 – how do the authors explain the observed difference between the interactions of G113 and K3593 of wt-CaM and N53I-CaM, respectively?
G113 is quite remote from N53? Is this difference really significant?
9. Page 7: "CaM-N53I and A102V alter spontaneous Ca2+ signalling events in cells." The HEK cells also have endogenous expression of CaM. How much additional CaM protein results from overexpression? So how much CaM variant to wt CaM is present in the experiments with N53I and A102V?
10. The authors should address the issue of dominance. If the affinity for the pathogenic CaM variants have lower affinity for RyR2 than, how can this lead to a dominant pathogenic effect? Recall that there are three CALM genes encoding identical CaM proteins, and thus only one of six alleles that carries the mutation.
11. It seems a bit odd that the authors choose to study and demonstrate an effect of (one of the) CaM mutations on CaMKIId phosphorylation without following up with biophysical investigation of the mechanism behind this.
Minor comments 1. Page 3, CALM genes encodes a 149 residue CaM protein, but the initiator Met is removed to generate the mature protein – text should be adjusted appropriately 2. Figure 1 – use same scale on y-axis for comparable plots (eg the ITC data)
3. The scale in figure 1c and d (right side) must be wrong.

---

**First revision**

Author response to reviewers' comments

First of all, the authors would like to thank the reviewers for their work. It is appreciated that the reviewers acknowledged the amount of work undertaken to characterize the CPVT- associated CaM variants and that they find the data novel and of high quality. This paper provides novel insights into the molecular mechanism of CaM-associated cardiac arrhythmias.

We would like to let the reviewers know that the title, abstract and number of displays have been amended to mee the journal requirements. All of the potential issues raised by the reviewers have been addressed below.

**Reviewer 1: Comments for the Author:**

**Major points.**
1. The amount of information the supplementary material was a distraction from the flow of reading as it required jumping back and forth between files. This was particularly annoying as many of the main manuscript figures have very little information and a lot of unoccupied white space (Figures 2, 3, 4, 5, 6 and 7, particularly later Figs). Most of the S figures could be included the main manuscript if the existing figures were rearranged and combined. Similarly tables could be combined and included in the main manuscript.
Considering the limit of 8 displays (figures + tables) required by the journal, we have redesigned the figures and included some of the supplementary data in the main manuscript. In addition, we have minimised empty spaces for all figures.

© 2021. Published by The Company of Biologists under the terms of the Creative Commons Attribution License (https://creativecommons.org/licenses/by/4.0/).

2. P3. Information/references about the CaM A102V and CaM-N53I mutations, previous studies: identification and human and model system studies. The two variants are named with no information apart from CPVT (without no reference). Please include this information.
We have added the following paragraph at the end of the introduction:
"CaM N53I variant was discovered in a large Swedish family with a severe dominantly inherited form of CPVT-like arrhythmias. Using a genome-wide linkage analysis, they demonstrated that the heterozygous missense mutation in the gene encoding calmodulin (*CALM1*) segregated with the disease and showed compromised calcium binding (Nyegaard et al., 2012). CaM A102V variant was identified in *CALM3* in a female who experienced episodes of exertion-induced syncope since age 10, had normal QT interval, and displayed ventricular ectopy during stress testing consistent with CPVT. CaM-A103V was shown to lower CaM $Ca^{2+}$-binding affinity and promoted spontaneous $Ca^{2+}$ wave and spark activity in permeabilised cardiomyocytes (Gomez-Hurtado et al., 2016)."

3. Discussion – how do you envisage the results could enlarge on.
To address this comment, we have added the following paragraph at the end of the Discussion: "In summary, both CPVT-associated variants CaM-N53I and CaM-A102V affect CaM:RyR2 structure-function relationship resulting in $Ca^{2+}$ release from SR, via unique molecular mechanisms. Based on our findings we propose that CaM-N53I likely acts through a loss of direct inhibition of RyR2 (subtle alterations in local structures), whereas for the CaM-A102V mutant, there is a finely tuned balance between loss of direct inhibition of RyR2 (higher Kd) and increased channel activation via CaMKII□ phosphorylation (Fig. 7). The loss of inhibition and over-activation of RyR2 triggers abnormal $Ca^{2+}$ release from the SR. The subsequent increase in cytoplasmic $Ca^{2+}$ concentration would then promote anomalous cardiac muscle contractions and generate irregular heartbeats, characteristics of CPVT syndrome. Interestingly, our data demonstrate a potential role of CaMKII□ in the molecular aetiology of the disease which could open ways for new therapeutic avenues."

**Minor.**

1. It is well known that EGTA is very acidic. Was the pH of the solution adjusted after addition of EGTA to the HEPES solution? Please clarify in the Results section on p.4 and in Methods.
EGTA was prepared as a stock solution (0.5 M with pH adjusted at pH 7.5 with KOH) and then added to the assay buffer (5 mM final). Because the pH of the EGTA solution was pre- adjusted, it did not affect the overall pH of the assay. For confirmation, we have used pH strips after adding the EGTA solution and the pH was still at 7.5 as expected.
To clarify this point in the manuscript, we have added the following statement in the Methods - Isothermal Titration Calorimetry (ITC) section:
"EGTA was prepared as a stock solution (0.5 M with pH adjusted to pH 7.5 with KOH) and then added to the assay buffer (5 mM final) to prevent any final pH changes."

2. P5, para 2, line 1 -typo "been" should be "being".
Text has been amended.

3. P8 line 4. Do you mean "previous studies" with the same variants?
To clarify this point, we have amended the text as follow:
"In accordance with previous studies investigating CaM-N53I, we showed that the secondary structure of the CaM-N53I variant was unchanged (Sondergaard et al., 2015a; Vassilakopoulou et al., 2015) and observed a reduced thermal stability (Sondergaard et al., 2015a), when compared to CaM-WT."

4. P9 para 4. Provide a reference for the clinical phenotypes mentioned.
A reference has been added.

**Reviewer 2: Comments for the Author:**

**Major concerns:**

1. The choice of length of the RyR2-CaMBD peptide used in this study could be problematic: in this manuscript, CaMBD2 ends at amino acid 3603 (the Phenylalanine involved in binding the N-lobe of CaM, thus introducing a negative charge from the C-terminal carboxylic acid in immediate

© 2021. Published by The Company of Biologists under the terms of the Creative Commons Attribution License (https://creativecommons.org/licenses/by/4.0/).

vicinity of the CaM binding). The published crystal structure of the CaM- RyR2(PDB 6Y4P) shows that the following three amino acids 3604-3607 (missing in this study) are quite close to parts of the N-lobe (amino acids 50, 51,54 and 71). Moreover, as the authors state, in the manuscript, a cryo-EM structure by Gong et al (2019) also showed that amino acids up to and including 3607 form the interaction site with CaM. Thus, the premature truncation at amino acid 3603 seems not an obvious choice and the premature truncation might be the cause for the differences in some of the results of this study and a previous study (Holt 2020): the previous study did not find any difference in affinity of CaM for CAMBD2 in the presence of Ca between the wt and the N53I variant (using amino acids 3581-3611)- this study does find a factor 3 different Kd (using amino acids 3583-3603). The authors comment on this discrepancy and explain it with the presence of Mg ions in their ITC experiment. It is not straightforward to see how 2mM Mg (which is free in solution) can cause a factor 3 drop in Kd of an interaction not involving Mg, while it is much more likely that a missing stretch of amino acids in CAMBD2, some of which close to the mutation site in CaM, would influence the Kd. The authors needs to address this. One option would be to demonstrate that the difference in Kd is maintained with a longer CaMBD2 peptide. If MG causes the difference, this should be easy to demonstrate (perform ITC without Mg).

The RyR2 peptide used in this study (3583-3603) has been shown to be the minimal binding domain for CaM (Tian et al., 2013; Yamaguchi et al., 2003). We understand that the new structural information available highlights 3 extra residues (3604-3607) as being involved in the interaction (Gong et al., 2019). In order to address this concern, we performed additional ITC experiments using a longer version of the peptide encompassing the additional residues, RyR23581-3608. We observed no significant difference in the affinity of CaM for RyR23583-3603 and RyR23581-3608 (figure below).

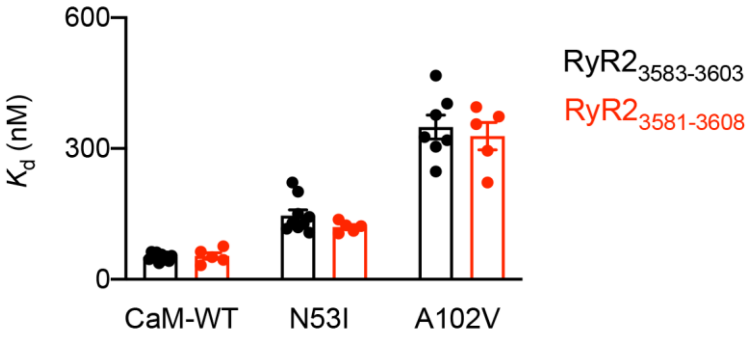


To determine whether the differences in $K_d$ observed for CaM-N53I when compared with Holt et al. (2020) were due to the presence of 2 mM $MgCl_2$, we performed additional ITC experiments in the absence of $MgCl_2$ (figure below). Our data demonstrate that $MgCl_2$ does not affect the binding of CaM to the peptide and we have removed this statement from the manuscript.

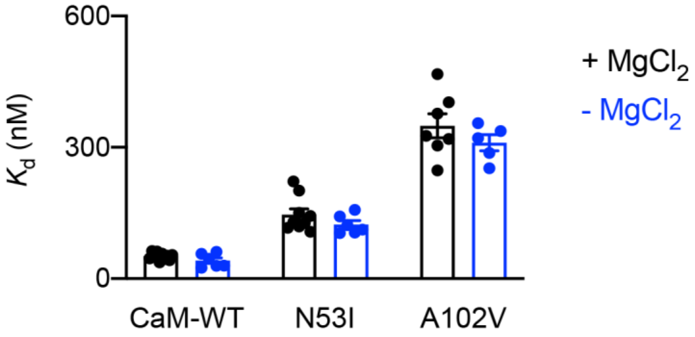


In conclusion, we have performed additional ITC experiments for all variants using 2 different peptides (RyR23583-3603 and RyR23581-3608) and in the presence/absence of magnesium. Altogether, we have now over 20 replicates confirming that the binding of CaM-N53I to RyR2 is significantly different from CaM-WT.
The above figures have been added to Supplementary materials (Fig. S5).

© 2021. Published by The Company of Biologists under the terms of the Creative Commons Attribution License (https://creativecommons.org/licenses/by/4.0/).

2. Paragraph entitled “the interaction of CaM with RyR2 is driven by the C-lobe of CaM”: Please mention, whether this paragraph deals with the Ca-free or Ca-bound version of CaM? (The methods section suggests that Ca was present, but it would be nice to have this clearly stated here, too. The observed “two-step” binding is very strange. If it were not for the ITC clearly yielding a 1:1 stoichiometry, those data would suggest a 2:1 stoichiometry. How would additional RyR2 free in solution impact on the structure of the already formed complex? With the given Kd values and concentrations, at 100 uM of both RyR2 and CaM, 98% of Cam should be bound to RyR. Moreover, both N and C-lobe change a lot of chemical shifts upon binding in the first step up to 1:1 ratio. The fact, that more C-lobe resonances shift than N-lobe resonances, cannot be used to argue that the “C-lobe drives the interaction”. Whether or not an interaction leads to a chemical shift change, depends on the nature of the interaction and on nearby amino acids. Nevertheless, the observed shift changes in the N-lobe upon continued addition of RyR2-CAMBD2 are not straightforward to understand.

This section in the Results has been re-written. The saturation of the CaM binding site and the plateauing of shift changes is dependent on the dissociation constant of the interactions. Hence, if the C-lobe binds stronger than the N-lobe, C-lobe resonances will saturate faster that the N- lobe resonances as the binding is an equilibrium with shifts changes indicating where you are in this equilibrium. We conclude that the C-lobe residues are more affected than the N-lobe at low peptide concentrations suggesting that the C-lobe forms the initial site of interaction.

3. P.8, end of 1st paragraph “[..] indicating that only the N53I variant induced significant structural changes.” This statement should be modified, since neither the results reported here not previous results by Holt et al show any indication of significant structural changes.
“significant” has been replaced by “subtle but notable”.

4. The abstract should thus also be modified to reflect point 3. The study does not provide evidence that the suggested subtle structural changes in N53I, when in complex with a truncated CaM binding peptide (which is contrasting the previously published by Holt et al) are the cause of RyR2 dysregulation.
A new version of the abstract has been created to reflect previous comments and to meet the word limit requirement for the journal.

5. The increased protease susceptibility of the N53I variant is more likely to come from an increase in local intramolecular dynamics, rather than a significant structural change. The authors should change the wording of their conclusions.
We would like to thank the reviewer for pointing this out, we have now amended the text accordingly.

“We demonstrated that conformational change adopted by CaM in the presence of $Ca^{2+}$ and/or RyR2 protected the protein against protease degradation (AspN), suggesting a significant change in local intramolecular dynamics of the protein. In all conditions tested, A102V susceptibility to proteases was similar to CaM-WT, indicating that only the N53I variant induced subtle but notable structural changes.” (2$^{nd}$ paragraph of the Discussion)

6. Page 8, 3rd paragraph: the two lobes of CaM are rather independent of each other and an intramolecular allosteric network between the two lobes would be really unusual. Moreover, the previous study by Holt et al did not report a significant shift change in any C-lobe amino acid upon mutating N53I.
The statement has been replaced by: “The NMR chemical shift perturbations of the variants show that in addition to the residues which are sequentially and spatially close the mutated residues being affected, more spatially distant residues also undergo shift perturbations. This indicates some intramolecular structural rearrangements of the tertiary structures in the variants.”

7. Page 8, last paragraph “We deposited the first high-resolution structures…”: in the strictest sense, the statement is correct, since no structure with the exact same amino acid range 3583-3603 has been published before. However, the statement is somewhat misleading, ignoring the structures presented by Holt et al. Modify appropriately.
We agree that Holt et al. have published their work before us. However, our statement remains correct, not because of the composition of the peptide, but because our structures were deposited

© 2021. Published by The Company of Biologists under the terms of the Creative Commons Attribution License (https://creativecommons.org/licenses/by/4.0/).

in PDB on the 27/01/2020 whereas theirs were deposited on the 21/02/2020. To avoid any confusion, we have removed the word “first” from the sentence.

8. Page 9 – how do the authors explain the observed difference between the interactions of G113 and K3593 of wt-CaM and N53I-CaM, respectively? G113 is quite remote from N53? Is this difference really significant?

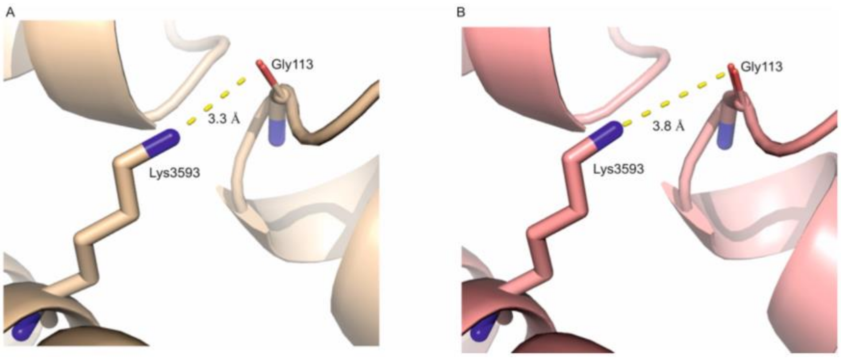


The CaM WT is represented in cartoon, beige (A) and CaM N53I, salmon (B). The distance between Gly113 from the protein and Lys3593 from the peptide is labelled and shown in dashed yellow lines. The difference in distance (3.8 Å> 3.3 Å) may be the reason for the lost H-bond interaction in $Ca^{2+}$/CaM N53I:RyR2 complex structure as the typical distance for H- bond formation is 2.7-3.3 Å. However, as this difference does not appear to be significant, the statement has been removed from the manuscript.

9. Page 7: “CaM-N53I and A102V alter spontaneous Ca2+ signalling events in cells.” The HEK cells also have endogenous expression of CaM. How much additional CaM protein results from overexpression? So how much CaM variant to wt CaM is present in the experiments with N53I and A102V?
We quantified CaM protein levels in HEK cells (untransfected or transfected with CaM mutants), using western blot. Protein levels (anti-CaM antibody) were quantified using densitometry (Fiji) and normalised to total protein levels (ponceau staining). We observed ~2- fold excess mutant protein expression, compared to endogenous CaM.

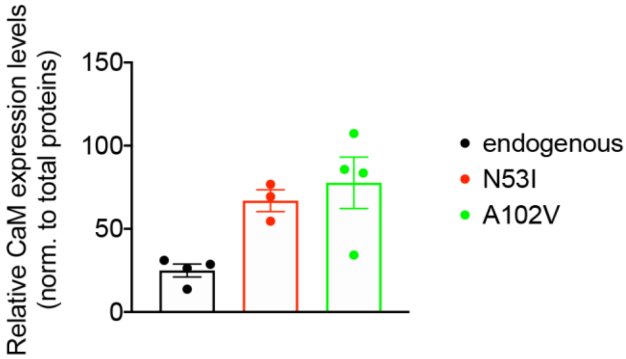


10. The authors should address the issue of dominance. If the affinity for the pathogenic CaM variants have lower affinity for RyR2 than, how can this lead to a dominant pathogenic effect? Recall that there are three CALM genes encoding identical CaM proteins, and thus only one of six alleles that carries the mutation.
The following paragraph has been added to the Discussion:
“In CaM-associated CPVT syndromes, only one out of six CaM alleles are mutated. However it has been shown that in the presence of 3-fold excess of CaM-WT, the variants CaM N53I and CaM-A102V can promote significantly higher $Ca^{2+}$ wave frequencies in permeabilized cardiomyocytes, when compared to CaM-WT (Hwang et al., 2014; Gomez-Hurtado et al., 2016). These data demonstrate that CPVT-associated CaM mutations can lead to a dominant pathogenic effect, which is consistent with an autosomal dominant inheritance pattern in humans. The functional dominance can be

© 2021. Published by The Company of Biologists under the terms of the Creative Commons Attribution License (https://creativecommons.org/licenses/by/4.0/).

explained by the fact that the tetrameric RyR2 $Ca^{2+}$ channel has four CaM binding sites and binding of only one single mutant CaM may be sufficient to disrupt the CaM-dependent regulation of $Ca^{2+}$ release from the SR."

11. It seems a bit odd that the authors choose to study and demonstrate an effect of (one of the) CaM mutations on CaMKIId phosphorylation without following up with biophysical investigation of the mechanism behind this.

In this paper, we demonstrate that CaM-A102V enhances CaMKII¤ phosphorylation activity (Fig. 5a). CaMKII is a complex enzyme composed of 12 subunits which can autophosphorylate and phosphorylate target substrates. To get a better understanding on the effect of CaM mutants on the molecular mechanism of CaMKII¤, we performed additional experiments focusing on the autophosphorylation of the enzyme ($Thr^{287}$).

Using western blot and densitometry analysis, we show that the CPVT-associated CaM mutants do not affect the rate of autophosphorylation (Fig. 5b), suggesting that CaM-A102V contribute to the increase of phosphorylation of the substrate.

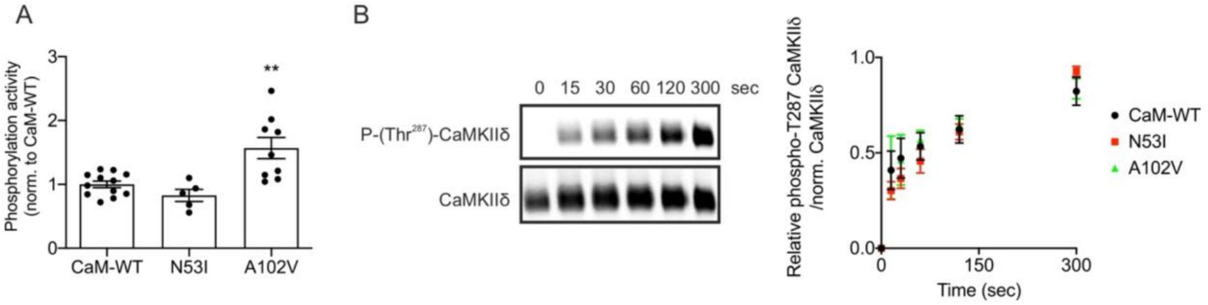


**Minor comments**

1. Page 3, CALM genes encodes a 149 residue CaM protein, but the initiator Met is removed to generate the mature protein – text should be adjusted appropriately

We agree that the CALM genes originally encode a 149 residue protein and that the initiator Met is removed to form the mature protein. For simplicity, it is well accepted to describe CaM as a 148 amino acid protein, as evidenced by publications from Profs Anthony Means and James Ames, leaders in the field of CaM.

2. Figure 1 – use same scale on y-axis for comparable plots (eg the ITC data)

Figure 1 has been redone and the same scale is used for similar plots.

3. The scale in figure 1c and d (right side) must be wrong.

Scale has been corrected by changing the unit in the axis title from kcal/mol to cal/mol.

---

Second decision letter

MS ID#: JOCES/2021/258796

MS TITLE: CPVT-associated calmodulin variants N53I and A102V dysregulate calcium signalling via different mechanisms

AUTHORS: Ohm Prakash, Marie Held, Liam F McCormick, Nitika Gupta, Lu-Yun Lian, Svetlana Antonyuk, Lee P Haynes, Nia Lowri Thomas, and Nordine Helassa
ARTICLE TYPE: Research Article

I am happy to tell you that your manuscript has been accepted for publication in Journal of Cell Science, pending standard ethics checks.

© 2021. Published by The Company of Biologists under the terms of the Creative Commons Attribution License (https://creativecommons.org/licenses/by/4.0/). 

Reviewer 1

*Advance summary and potential significance to field*

As summarised by the authors, they provide high-resolution 3D structures of CaM variants in complex with RyR23583-3603 and novel data on the binding mechanism of CaM to RyR2. They show for the first time that arrhythmogenic variants N53I and A102V alter intracellular Ca2+ oscillation kinetics and can significantly increase CaMKIIδ phosphorylation activity. Collectively, these data provide novel insight into the molecular aetiology of CPVT with abnormal Ca2+ release from SR via distinct molecular mechanisms for N53I and A102V.
These findings are significant in understanding how structural changes in CaM variants alter intracellular Ca2+ signalling to produce a CPVT phenotype.

*Comments for the author*

My previous comments have been adequately addressed by the authors.

I have no further comments.

Reviewer 2

*Advance summary and potential significance to field*

This paper demonstrates that CPVT associated A102 calmodulin variant does not induce significant structural changes when bound to the RyR2 binding peptide at high Ca2+. This is similar to the subtle structural effect observed in the CPVT associated N53I calmodulin variant. These observations are interesting and leaves the field with the notion that RyR2 regulation is extremely sensitive to the slightest variation in the regulating calmodulin protein. The study demonstrates in contrast to N53I, the A102V calmodulin variant leads to increased CaMKIId phosphorylation. This activity is novel and demonstrates that there may be significant differences in the molecular pathogenic mechanisms for the rare individual calmodulin mutations, despite the occurrence of seemingly identical phenotypes.

*Comments for the author*

The authors have responded in a satisfactory manner to the questions raised in the initial review. I still think the title is over stating the impact of the data shown. The authors confirm that both N53I and A102V mutations dysregulate RyR2 activity demonstrated in prevoius studies by oher groups. However, the manuscript does not provide conclusive data for the mechanism of RyR2 activity dysregulation. They are more of a suggestive nature. Thus I recommend adjusting the title and abstract accordingly.

© 2021. Published by The Company of Biologists under the terms of the Creative Commons Attribution License (https://creativecommons.org/licenses/by/4.0/).
